# Measuring the performance of prediction models to personalize treatment choice

**DOI:** 10.1002/sim.9665

**Published:** 2023-01-26

**Authors:** Orestis Efthimiou, Jeroen Hoogland, Thomas P.A. Debray, Michael Seo, Toshiaki A. Furukawa, Matthias Egger, Ian R. White

**Affiliations:** 1 Institute of Social and Preventive Medicine (ISPM), University of Bern, Bern, Switzerland; 2 Institute of Primary Health Care (BIHAM), University of Bern, Bern, Switzerland; 3 Department of Psychiatry, University of Oxford, Oxford, UK; 4 Julius Center for Health Sciences and Primary Care, University Medical Center Utrecht, Utrecht University, Utrecht, The Netherlands; 5 Department of Epidemiology and Data Science, Amsterdam University Medical Centers, Amsterdam, The Netherlands; 6 Smart Data Analysis and Statistics B.V., Utrecht, The Netherlands; 7 Graduate School for Health Sciences, University of Bern, Bern, Switzerland; 8 Departments of Health Promotion and Human Behavior and of Clinical Epidemiology, Kyoto University Graduate School of Medicine/School of Public Health, Kyoto, Japan; 9 Centre for Infectious Disease Epidemiology and Research, Faculty of Health Sciences, University of Cape Town, Cape Town, South Africa; 10 Population Health Sciences, Bristol Medical School, University of Bristol, Bristol, UK; 11 MRC Clinical Trials Unit at UCL, University College London, London, UK

**Keywords:** prediction modelling, personalized medicine, heterogeneous treatment effects

## Abstract

When data are available from individual patients receiving either a treatment or a control intervention in a randomized trial, various statistical and machine learning methods can be used to develop models for predicting future outcomes under the two conditions, and thus to predict treatment effect at the patient level. These predictions can subsequently guide personalized treatment choices. Although several methods for validating prediction models are available, little attention has been given to measuring the performance of predictions of personalized treatment effect. In this article, we propose a range of measures that can be used to this end. We start by defining two dimensions of model accuracy for treatment effects, for a single outcome: discrimination for benefit and calibration for benefit. We then amalgamate these two dimensions into an additional concept, decision accuracy, which quantifies the model’s ability to identify patients for whom the benefit from treatment exceeds a given threshold. Subsequently, we propose a series of performance measures related to these dimensions and discuss estimating procedures, focusing on randomized data. Our methods are applicable for continuous or binary outcomes, for any type of prediction model, as long as it uses baseline covariates to predict outcomes under treatment and control. We illustrate all methods using two simulated datasets and a real dataset from a trial in depression. We implement all methods in the R package predieval. Results suggest that the proposed measures can be useful in evaluating and comparing the performance of competing models in predicting individualized treatment effect.

## 1 Introduction

Clinical prediction models are an important tool in modern clinical practice.^1^ Typically, prediction models use a range of patient-level covariates (also called predictors or prognostic factors) to forecast the future outcome. Classical statistical techniques can be used to develop such models, while machine learning methods have become increasingly popular in recent years, especially for analyzing the so-called Big Data.^2,3^ There is, however, an ongoing debate on whether (or rather, under what circumstances) machine learning brings any added benefit in practice.^4,5^ Most applications of clinical prediction models aim to predict absolute values (for continuous outcomes) or probabilities of an event (for binary outcomes), and are not usually designed for comparing outcomes under different treatment regimes, that is, are not aimed at evaluating treatment effects such as a risk difference.

The decision between treatments in clinical practice and recommendations in guidelines are predominantly informed by results from randomized clinical trials (RCTs) or meta-analyses of RCTs, using estimates of the average relative treatment effect to decide on how to treat new patients. However, there is increasing recognition that a treatment may provide different absolute benefits to different patients. For example, the genetic characteristics of patients, their demographic characteristics, and the severity of their disease, may all modify the difference in risk of an event between two treatments (and this might happen even if the relative risk remains constant). Treating patients who will gain only small or even zero benefit may lead to unnecessary costs or avoidable side effects. Thus, the common one-size-fits-all approach to treating patients based on the average treatment effect may be wasteful or even harmful. For these reasons, a “stratified”—or even “personalized”—approach to treating patients is of great interest.^6,7^ To achieve this, there is mounting interest in clinical prediction models that the forecast patients’ outcomes under different treatment conditions. Rekkas et al. provided recently a review of relevant methods.^8^ Such models provide patient-level estimates of treatment benefit or harm, thus supporting personalized clinical decision-making.^9^

Further, a model that works well in predicting the outcome might not perform well when estimating treatment benefit. Indeed, minimizing the mean squared error of outcome predictions does not necessarily minimize the error of the treatment benefit predictions.^10,11^ This motivated the development of methodologies that directly focus on benefit, rather than on predicting absolute outcomes.^10,12,13^ However, the problems of assessing model performance and performing model selection remain. More specifically, while it is relatively straightforward to assess the performance of a model for predicting an outcome (eg, by comparing observations with predictions), it is more difficult to assess model performance when the main interest is in treatment benefit. This is because in most study designs, we only observe the outcome under a single treatment for each patient, which means that the treatment benefit at the patient level is unobservable.^14^ This is related to the “fundamental problem of causal inference.”^15^ In the recent years, some approaches have been proposed for assessing a model’s capacity to predict treatment benefit^10,11,16,17^; Schuler et al. provide an overview^18^; in the recent preprint Maas et al. described additional methods.^19^

Here, we build on the previous work and present a range of methods for evaluating and comparing models with respect to their ability to predict patient-level treatment effect for a single outcome. Our starting point is to define two dimensions of accuracy of individualized treatment effect predictions, that is, *discrimination for benefit* and *calibration for benefit*. In addition, we propose a new concept, *decision accuracy*, which quantifies the ability of the model to identify patients whose treatment benefit exceeds a threshold, *B*_*Th*_. Next, we propose relevant performance measures and present a range of estimating procedures. Any statistical or machine learning method for predicting a binary or a continuous outcome can be accommodated by our framework, as long as it provides a prediction of the outcome under treatment and control, given a set of patient baseline predictors. We focus on model evaluation in randomized data and briefly discuss possible extensions to observational data. We illustrate all methods using a simulated dataset and we provide an R package that can be used to apply all our methods. We implement the new methods using a real dataset obtained from a large trial comparing pharmacotherapies for people with depression. Finally, we discuss how our measures can be embedded in a decision-making process, where we usually must consider multiple effectiveness and safety outcomes as well as costs.

## 2 Notation

We start by presenting all notation used in this article. We assume that our dataset includes *N*_*p*_ patients and a complete set of baseline covariates. We assume that patients were randomized to treatment or control (*t*_*i*_ = 1/0) and that we observe outcome *y*_*i*_ (binary or continuous) for patient *i*. Next, for the case when *y*_*i*_ is continuous, we assume that there are underlying true outcomes under treatment and control for each patient. The difference between these individual “counterfactual” outcomes (ie, treatment minus control) is the true treatment benefit *B*_*i*_, which we are primarily interested in estimating. Likewise, when *y*_*i*_ is binary, we assume that the occurrence of an event is stochastic, and that the probability of an event may differ between the treatment and control condition. The treatment benefit *B*_*i*_ is the difference between the latter event probabilities (ie, risk difference). Note that *B*_*i*_ is inherently unobservable since each patient only received one treatment in this setting. For both continuous and binary outcomes, we assume that a patient for whom *B*_*i*_ > *B*_*Th*_ would benefit from treatment rather than control with respect to this particular outcome; the opposite for *B*_*i*_ < *B*_*Th*_. Here, *B*_*Th*_ is a benefit threshold, that is, a value for *B*_*i*_ above which treating patients is worthwhile. In our examples below we consider the case of *B*_*Th*_ = 0 for simplicity. A nonzero value for *B*_*Th*_ would be the relevant when the treatment is associated with costs or side effects. Next, we develop a model *M* using all observed data from treated and control individuals. This can be any statistical or machine learning model. Given the patient’s covariates, for a continuous outcome, model *M* predicts outcomes under treatment(*ŷ*_*iM,t*=1_) and control(*ŷ*_*iM,t*=0_), and thus treatment benefit ${\hat{B}}_{iM}={\hat{y}}_{iM,t=1}-{\hat{y}}_{iM,t=0}.$ Likewise, for binary outcomes we estimate probabilities ${\hat{p}}_{iM,t=1}$ and ${\hat{p}}_{iM,t=0},$ and benefit ${\hat{B}}_{iM}={\hat{p}}_{iM,t=1}-{\hat{p}}_{iM,t=0}$ on the risk difference scale. The interest in this article is on methods for comparing *B*_*i*_ with ${\hat{B}}_{iM},$ using observed *y*_*i*_ and covariates. Table 1 provides an overview of all notation used in this article.

## 3 Motivating Datasets

We illustrate the proposed methodology using a simulated dataset for a continuous outcome and a real example including both continuous and binary outcomes. In the appendix, we also provide an example of a simulated dataset for a binary outcome.

### 3.1 Simulated dataset

The dataset included 1000 patients, where for each patient *i* we had four patient-level covariates (*x*_*i*1_, … *x*_*i*4_). Patients were randomized to *t*_*i*_ = 1 or *t*_*i*_ = 0 with 50% probability. The outcome *y*_*i*_ was continuous and was generated using terms linear to *x*_*i*1_, *x*_*i*2_, *x*_*i*3_, *x*_*i*4_, and *t*_*i*_, and also including some interactions between covariates and interactions between treatment and *x*_*i*1_, *x*_*i*3_. We assumed larger values of *y*_*i*_ to be preferable (eg, reflecting a patient-reported outcome such as quality of life), while we also generated the counterfactual outcome, to allow us to evaluate the performance of our performance metrics. Full details are provided in the appendix. The mean observed outcome was 5.17. According to the data-generating mechanism, 773 patients in this sample would benefit from taking *t* = 1 and 227 from *t* = 0. The mean true benefit was 0.29.

To analyze these data, we developed two simple regression models. The first (*M*_1_) was defined as *y*_*i*_~*x*_*i*1_ + *x*_*i*3_ + *t*_*i*_ + *x*_*i*1_ *t*_*i*_ + *x*_*i*3_ *t*_*i*_. This model was misspecified with respect to the data-generating mechanism and did not include several genuine predictors but included the correct treatment-covariate interactions. The second model, *M*_2_ was also mis-specified; it included more genuine predictors than *M*_1_, but missed an important interaction between treatment and *x*_*i*3_: *y*_*i*_~*x*_*i*1_ + *x*_*i*2_ + *x*_*i*3_ + *x*_*i*4_ + *t*_*i*_ + *x*_*i*1_ *t*_*i*_ + *x*_*i*2_ *t*_*i*_. These two “incorrect” models were prespecified for illustration purposes; data-driven methods (eg, based on LASSO) would probably lead to other model specifications. We use this example here to show how a model that predicts the observed outcome well may fail to capture treatment benefit; in the later sections we use it to illustrate the proposed measures. We estimated both models in the dataset of 1000 patients. We first compared the models’ ability to predict the absolute outcomes. We compared predictions *ŷ*_*i*_ from each model with the ground truth *y*_*i*_, and calculated the root mean squared error (RMSE) as an overall measure of model accuracy. We also fit a linear regression *y*_*i*_*~ŷ*_*i*_ to obtain the coefficient of determination (*R*^2^). We found that *M*_2_ performed better than *M*_1_ in terms of RMSE (1.08 for *M*_1_ vs 1.03 for *M*_2_) and *R*^2^ (0.26 vs 0.33). We also found superior performance of *M*_2_ when comparing AIC (3010 for *M*_1_, 2918 for *M*_2_) and BIC (3044 vs 2962). These results indicate that *M*_2_ is preferable over *M*_1_ for outcome risk prediction and might thus be taken to suggest (naively) that *M*_2_ should also be preferred for prediction of treatment benefit.

However, opposite conclusions were drawn when evaluating treatment benefit predictions of *M*_1_ and *M*_2_. Using the models, we predicted treatment benefit ${\hat{B}}_{iM}$ for each patient and for each model, and compared it with the true underlying benefit *B*_*i*_ by calculating the RMSE, and by fitting the regression $B_{i}\sim {\hat{B}}_{iM}.$ Results were: RMSE 0.12 for *M*_1_ vs 0.42 for *M*_2_; slope 1.08 vs 1.24; *R*^2^ 0.95 vs 0.32. Note that these results were not affected by overfitting: after using the developed models to make predictions in the new sample of 10 000 we got similar results for all performance measures (of course, in real applications such a big external sample would often not be available). Thus, *M*_2_ performed much worse than *M*_1_, that is, although *M*_2_ outperformed *M*_1_ in terms of absolute outcome prediction (ie, prediction of *y*_*i*_ given treatment choice and covariates), it was inferior to *M*_1_ for predicting personalized treatment benefit. This apparent contradiction was expected, since *M*_2_ did not include the correct treatment-covariate interactions to estimate treatment benefit. This example shows that a model may perform well for absolute outcome predictions (ie, when treatment choice is already made), but badly for predicting absolute treatment effect (ie, when treatment choice is not yet established). It also shows that choosing a model according to its ability to predict the outcome may lead to selecting models that fail to capture patient-level treatment benefit.

### 3.2 Case study: Antidepressant treatment of patients with unipolar major depression

The dataset was obtained from SUN😊D (Strategic Use of New generation anti-depressants in Depression). This was a two-step multi-center trial comparing first- and second-line treatment strategies for patients with unipolar major depression. At Step 1 of the trial, all participants received sertraline. At Step 2, participants who were not in remission by week 3 were randomized to continue sertraline, to add mirtazapine to sertraline, or to switch to mirtazapine. Remission was defined as scoring 4 or less on the Personal Health Questionnaire-9, PHQ-9. PHQ-9 ranges from 0 to 27, where higher values indicate more severe symptoms. The study was powered to detect an overall treatment effect in depression symptoms measured at week 9.^20^

We used the data from arms 2 and 3 of the second step, that is, patients randomized to sertraline and mirtazapine (*N* = 502) and patients randomized to switch to mirtazapine (*N* = 530). The first outcome we used was symptom severity at week 9, measured as total PHQ-9 score. The second outcome was remission (PHQ-9 ≤ 4). Of note, dichotomizing a continuous outcome is usually a bad idea, as it leads to loss of information.^21^ We did it here, however, for illustration purposes. The dataset also contained many patient-level covariates including socio-demographic variables (age, sex, education in years, employment and marital status), and depression-related variables (age at onset, number of previous episodes, length of index episode, and concurrent physical conditions). It also included the item scores of the PHQ-9 at week 1 and 3 (ie, the time of randomization), and the Beck Depression Inventory-II (BDI-II) at weeks 1 and 3, and the Frequency Intensity and Burden of Side Effects Rating (FIBSER) at week 1 and 3. The baseline PHQ-9 and FIBSER score were prespecified for subgroup analyses in the original protocol.^20^

Missing data were not a source of concern for this dataset: only 4% of the patients had missing values for one or more predictors or outcomes. For simplicity, we limited our analyses to patients with complete data. The results of the main analyses have been published elsewhere.^22^ This dataset was later re-used to develop a set of prediction models for the outcome under the various treatments, to facilitate a personalized choice of treatments.^23^ The data cannot be made publicly available due to confidentiality agreements.

## 4 Methods

### 4.1 General concepts and definitions

#### 4.1.1 Discrimination and calibration when predicting outcomes

When developing a clinical prediction model, it is recommended to assess its performance in terms of calibration and discrimination.

*Discrimination* is the ability of the model to correctly rank-order patients with respect to their outcomes. For example, for a continuous outcome, if we use a model with perfect discrimination, among two randomly chosen patients, the one with the higher predicted outcome will also have the higher observed outcome. This can be examined using rank correlation statistics between predictions and observations. For a binary outcome, discrimination relates to the ability of a model to split the population into groups at different risks. The area under the receiver operating characteristic curve (AUC) is a standard measure of discrimination for binary outcomes and is equal to the concordance statistic.

*Calibration* refers to the agreement between observed outcomes and the model’s predictions. For continuous outcomes, calibration can be examined via a scatterplot and by fitting a calibration line, that is, regressing observed predicted. For binary outcomes, calibration is usually quantified using the ratio of observed vs predicted events, the calibration intercept, and the calibration slope. For example, for a well-calibrated model, among patients for whom we predicted 30% probability of an event, 30% experienced the event.^1^

Note that other common measures of performance for continuous outcomes, such as *RMSE, R*^2^ and mean absolute error, combine calibration and discrimination aspects, that is, they measure overall accuracy.

#### 4.1.2 Dimensions of model accuracy when predicting treatment benefit

This article focuses on methods for predicting treatment effect rather than the outcome per se. Hence, measures of model accuracy should quantify the model’s ability to predict treatment effect. For example, for a binary outcome, the focus is on the reduction in risk associated with a treatment, rather than the overall risk of experiencing the outcome. Thus, we propose the following definitions:

*Discrimination for benefit*: the ability of a model to rank-order patients with respect to the benefit they would receive from treatment. For a perfectly discriminating model *M*, for two patients *i, j* for whom *B*_*i*_ > *B*_*j*_, then ${\hat{B}}_{iM}>{\hat{B}}_{jM}.$ In other words, discrimination for benefit relates to the ability of a model to differentiate patients who will benefit more from patients who will benefit less from treatment.

*Calibration for benefit*: the agreement between predicted and true treatment effects. For example, for a model well-calibrated for benefit, among patients for which we predicted X amount of benefit (on some scale), the true benefit is indeed X.

A model’s ability to predict individual treatment effects combines these two dimensions. A model with good discrimination for benefit might not be well calibrated for benefit; for example, a model may perfectly identify patients who would benefit more from a treatment, but at the same time, may overestimate the effect of the treatment. Likewise, there may be a model that is well-calibrated on average (eg, patients have accurately predicted benefit from 2 to 3%) but may fail to identify which patients among them would gain more benefit from treatment; in this case the model would have good calibration but bad discrimination for benefit.

Moreover, as also noted by Fernández-Loría and Provost,^17^ a model that is optimal in predicting treatment benefit may be suboptimal for making treatment decisions. Thus, we additionally define:

*Decision accuracy*: the ability of a model to identify patients who would benefit by at least *B*_*Th*_ from receiving treatment (*t* = 1) rather than control (*t* = 0). If we choose the threshold to be *B*_*Th*_ = 0, a model with perfect decision accuracy would maximize overall benefit in the population (with respect to this particular outcome), by identifying patients who should be given treatment (*B*_*i*_ > 0) and patients who should be given control (*B*_*i*_ < 0). Alternatively, when choosing a positive value of *B*_*Th*_ (eg, to reflect possible side effects of the treatment), a perfect model would maximize the overall risk–benefit tradeoff.

Decision accuracy combines aspects of discrimination and calibration for benefit. More specifically, it requires discriminative performance around a relevant decision threshold (where the model should be well calibrated). In what follows we assume *B*_*Th*_ = 0, but a generalization to different thresholds is straightforward. We refer our readers to the Discussion section for additional considerations regarding aspects of prediction accuracy with respect to treatment effect.

#### 4.1.3 Internal validation of prediction models

In the following sections, we discuss measures for assessing model performance with respect to the dimensions described above. In describing these measures, we will assume that we have first obtained out-of-sample predictions of treatment benefit for all patients. An easy way to do this is via a *k*-fold cross-validation (CV). More details are given in Section 1 of the appendix.

### 4.2 Assessing discrimination for benefit: C-for-benefit

A method for discrimination for benefit developed for binary outcomes was proposed by van Klaveren et al.,^24^ the so-called C-for-benefit. To calculate C-for-benefit, we first create pairs of the “similar” patients, one of whom received treatment, the other control. Then, for each pair we measure observed benefit. Quoting from the article, C-for-benefit measures the probability that “from two randomly chosen matched pairs with unequal observed benefit, the pair with greater observed benefit also has a higher predicted benefit”. The authors proposed two methods for matching patients, that is, using either their covariates or the predicted benefit from the model. Unfortunately, the target estimand of this approach is not clearly defined. Different choices with respect to the matching method may affect both the estimates and “true values” (ie, the estimand) of the performance measure; thus C-for-benefit results can be ambiguous. Note that other methods presented below may also use matching as part of the estimation procedure, but the estimand is not affected. In the recent article by Hoogland et al. (still a preprint by the time this text is written),^25^ the authors evaluated the limitations of C-for-benefit in more detail and proposed a new measure for discrimination, the model-based c-for-benefit. However, we do not discuss this here in more detail.

### 4.3 Assessing calibration for benefit

The performance measures of interest are the mean bias, that is, $E\left(B_{i}-{\hat{B}}_{iM}\right)$ (which is analogous to “calibration in the large” for usual prognostic models^26^), the intercept (*a*_0_) and slope (*a*_1_) of the line $B_{i}\sim {\hat{B}}_{iM}$ in the population. Ancillary summary measures of interest are the mean squared error $RMSE=\sqrt{E\left({\left(B_{i}-{\hat{B}}_{iM}\right)}^{2}\right)}$ and the *R*^2^ of the $B_{i}\sim {\hat{B}}_{iM}$ line. Of note, RMSE and *R*^2^ measure overall performance, rather than just calibration.

The estimation of mean bias is straightforward for a continuous outcome, assuming randomization: we compare the observed mean benefit at the arm level (mean observed outcome in treatment minus control) to the mean-predicted benefit. For binary outcomes we can easily compare the observed risk difference at the arm level to the average predicted benefit, calculated as the average predicted probability of an event for patients in *t* = 1 minus average predicted probability in *t* = 0.

The next sections describe ways to estimate remaining performance measures.

#### 4.3.1 Grouping according to predicted benefit

Given that the model predictions were obtained using a *k*-fold CV procedure (Section 1 of the appendix), all ${\hat{B}}_{iM}$ have been computed from model parameters estimated outside the present data. In other words, ${\hat{B}}_{iM}$ is in effect a baseline covariate for each patient. This allows us to perform subgroup analyses based on it, because this way randomization will remain intact, meaning that we can readily estimate causal effects of treatment.

Specifically, we split patients into *N*_*g*_ groups, according to ${\hat{B}}_{iM}.$ Given that the treatment assignment is randomized, each group will in principle include both treated and untreated individuals, and we can estimate the observed treatment benefit within each group as the mean outcome in patients on *t* = 1 minus that in patients on *t* = 0. We calculate the average predicted benefit within each group as discussed in the previous paragraph. Finally, we fit a line to the observed-predicted benefit pairs, to estimate all quantities of interest. Furthermore, we can visualize results in a scatterplot. We can add a regression line to the plot (or even a smooth curve if *N*_*g*_ is large). We can also add bars showing the standard errors of the observed benefit (*y*-axis) and the SD of the predicted benefit (*x*-axis). This approach may allow us to find groups of patients for whom the model correctly identifies large benefits or harms from treatment, which can be particularly important for decision-making.

#### 4.3.2 Clustering using covariates

Another approach is to try to create groups of “similar” patients, and then compare mean observed vs predicted benefit at the group level (ie, group-level approximations of *B*_*i*_ and ${\hat{B}}_{iM}$ respectively), to estimate all measures of interest. More specifically, we use patient covariates and an unsupervised clustering algorithm (eg, k-means) to group patients into *N*_*g*_ groups. Within each group we estimate observed and predicted benefit as above, and we compare the two sets of values to estimate all measures. Given that the clustering procedure may lead to different groups each time it is executed (k-means depends on an arbitrary choice of initial centroids), we repeat the procedure multiple (eg, 1000) times and then average, to obtain stable results.

One disadvantage of this method is that it is heavily dependent on the clustering procedure, and the covariates used for it. For example, if across the groups there is not enough variation in the true or the predicted benefit, this method will fail. Thus, the method of Section 4.3.1 might be preferable. Note that instead of clustering into groups of multiple patients, we could use a matching method that is, match patients one-on-one, and repeat multiple times. We provide more details in the appendix.

#### 4.3.3 Regression for benefit

A different approach to assess calibration for benefit is to regress the observed outcome on the treatment assignment and the predicted treatment benefit. The quantity we try to estimate here is the slope of the $B_{i}\sim {\hat{B}}_{iM}$ line, where a good-performing model will have a slope close to 1. To estimate, for a continuous outcome we fit the following regression on the observed data: $y_{i}\sim b_{0}+b_{1}{\hat{y}}_{iM,t=0}+b_{2}t{\hat{B}}_{iM},$ where, *b*_2_ = 1 suggest a model with perfect calibration for benefit. The intuition behind this approach is that we want to separate the ability of the model to predict outcomes in *t* = 0 from its capacity to estimate treatment benefit; as we mentioned already, a model might work well for the former but might underperform for the latter. For a binary outcome we fit instead a logistic regression model logit $\left(p_{iy}\right)\sim b_{0}+b_{1}\text{logit}\left({\hat{p}}_{iM,t=0}\right)+b_{2}\;t\;{\hat{B}}_{iM},$ where ${\hat{B}}_{iM}$ should be here in the log-odds ratio scale. Another alternative at this point would be to fix the *b*_1_ coefficient in the two models above to be equal to 1 (ie, to have *ŷ*_*iM,t*=0_ or logit $\left({\hat{p}}_{iM,t=0}\right)$ as offset terms), as discussed by Hoogland et al.^25^ However, the choice between these two alternatives is not straightforward, and it would require simulations; see also the Discussion.

### 4.4 Assessing decision accuracy

#### 4.4.1 Population benefit: dichotomizing by predicted benefit

**Figure 1:**
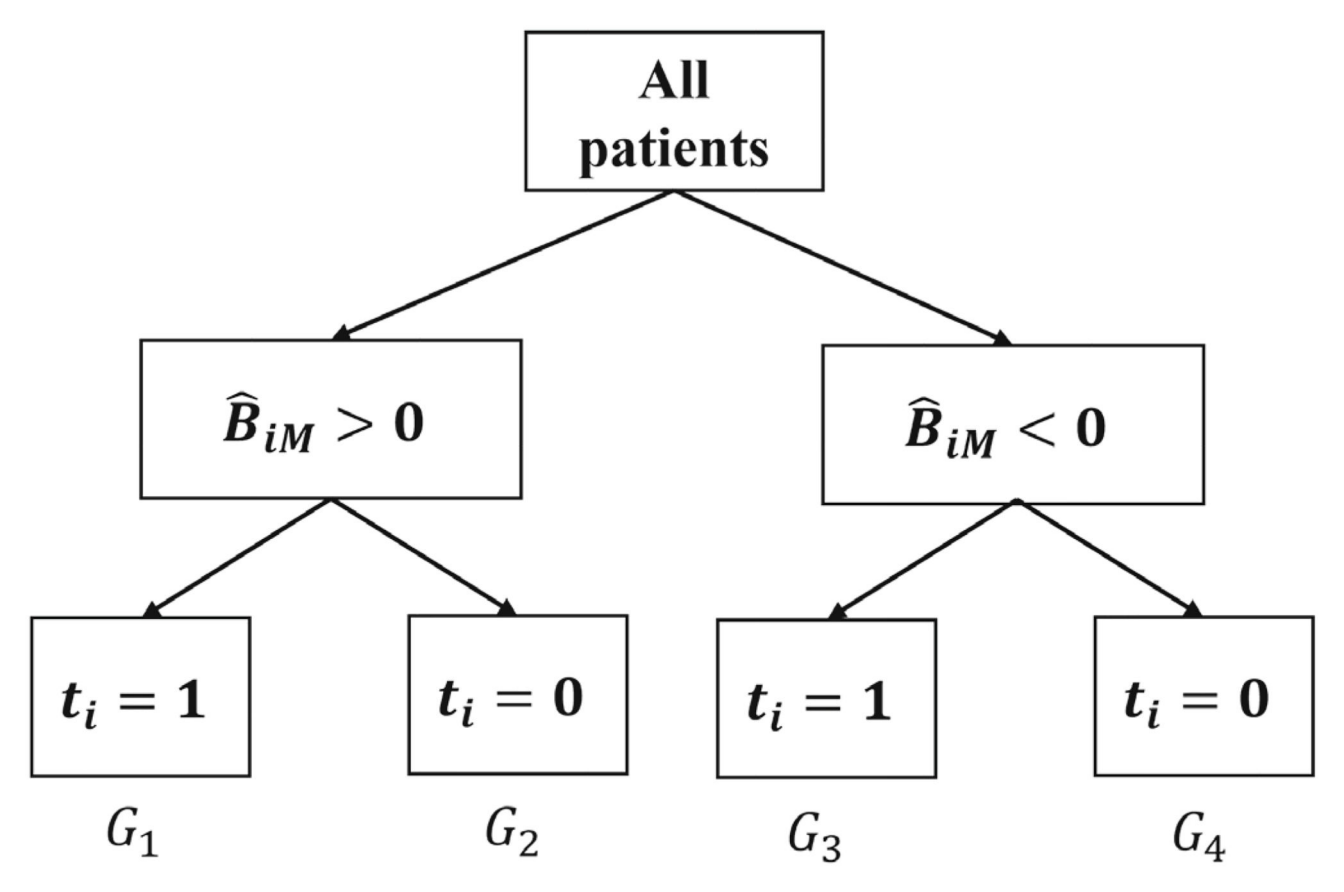
Schematic representation of how patient *i* is assigned into a group according to predicted treatment benefit (${\hat{B}}_{iM}$) from model *M*, and treatment received (*t*_*i*_ = 0/1). Patients in groups *G*_1_ and *G*_4_ received the optimal treatment according to model *M*, patients in *G*_2_ and *G*_3_ the suboptimal. Here we have assumed treatment benefit threshold to be zero, that is,. *B*_*Th*_ = 0.

Our first measure of decision accuracy assesses the average benefit of using a prediction model *M* to decide to treat patients, assuming a threshold *B*_*Th*_ = 0. We define the population-level benefit as the difference between the mean expected outcome if we followed the recommendations of model *M* (ie, we give *t* = 1 to patient *i* when ${\hat{B}}_{iM}>0$; *t* = 0 when ${\hat{B}}_{iM}<0$) vs the mean expected outcome if we followed the opposite of what *M* recommended. A model with perfect decision accuracy will maximize population-level benefit, by identifying the better treatment for each patient. The (model-specific) performance measure of interest is

(1) $$PB_{M}=E(y|\text{treat}\;\text{according}\;\text{to}\;\text{model}\;M)-E(y|\text{treat}\;\text{with}\;\text{the}\;\text{opposite}\;\text{of}\;\text{model}\;M)$$

where *PB* stands for “population-level benefit.” To estimate ${\hat{PB}}_{M},$ we use ${\hat{B}}_{iM},$ to split patients into four groups as shown in Figure 1. Patients in group *G*_1_ (*G*_4_) were treated with *t* = 1 (*t* = 0), and according to *M* they received their optimal treatment. Patients in group *G*_2_ (*G*_3_) were treated with *t* = 1 (*t* = 0), and according to *M* they received their suboptimal treatment. Then, we can estimate *PB*_*M*_ as follows:

(2) $${\hat{PB}}_{M}={\bar{y}}_{G_{1}UG_{4}}-{\bar{y}}_{G_{2}UG_{3}}=\frac{\sum_{i\in G_{1}}y_{i}+\sum_{i\in G_{4}}y_{i}}{n_{1}+n_{4}}-\frac{\sum_{i\in G_{4}}y_{i}+\sum_{i\in G_{3}}y_{i}}{n_{2}+n_{3}},$$

where *n*_1_, …, *n*_4_ denotes the number of patients in *G*_1_, …, *G*_4_. Because of randomization and because ${\hat{B}}_{iM}$ is in effect a baseline covariate (due to how it was estimated, that is, via *k*-fold CV), Equation (2) is an unbiased estimate of *PB*_*M*_. However, we can improve estimation by accounting for possible imbalance in covariates.^27^ To do so, we create a dummy variable *agree* for each patient, where *agree* = 1 if a patient is in *G*_1_ ∪ *G*_4_ (ie, treatment assignment agrees with model recommendation), 0 otherwise. Note that expression (2) is just the mean difference between the subgroups *agree* = 1 and *agree* = 0. Then, for a continuous outcome we regress the observed outcome over *agree*, also including all other observed covariates in the regression. Then, we take the coefficient of *agree* as the estimate ${\hat{PB}}_{M}.$ Note that instead of a regression adjustment we could follow inverse probability of treatment weighting.^28^ In this case, *agree* would assume the role of “treatment.” For a binary outcome, we can estimate ${\hat{PB}}_{M}$ as a marginal risk difference. We first fit a logistic regression model on the observed outcome over *agree*. Then, we use the fitted model to estimate the probability of an event for all patients in the dataset after setting *agree* = 1, and then after setting *agree* = 0. The mean difference between the two is our covariate-adjusted ${\hat{PB}}_{M}.$ To obtain confidence intervals of this estimate we can either use the so-called standardization method (also termed “marginalization” or “G-computation”)^27^ or we can use bootstrapping. Of note, bootstrapping here does not account for model estimation. In addition, the division of patients in the four groups shown in Figure 1 is only possible if there are both patients with positive and negative predicted benefit. If all patients have positive or all patients have negative estimated benefit, the calculation of *PB* using Equation (1) becomes trivial, as it just equals the average treatment effect. In that case, this analysis is more meaningful if we assume nonzero benefit threshold.

Other performance measures can be used instead of the *PB*_*M*_ defined in Equation (1). For example, we could compare outcomes when following the model, vs treating no-one, that is, *t* = 0 for all patients. Then, the performance measure would be

$$PB_{M}^{\left(0\right)}=E(y|\text{treat}\;\text{according}\;\text{to}\;\text{model}\;M)-E(y|t=0).$$

Likewise, we could compare outcomes after following the model vs after treating everyone, with the performance measures being

$$PB_{M}^{\left(1\right)}=E(y|t\text{reat}\;\text{according}\;\text{to}\;\text{model}\;M)-E(y|t=1).$$

Arguably these two performance measures are clinically more intuitive than *PB*_*M*_ defined in Equation (1), and may be more useful for evaluating the absolute performance of a model. When it comes to comparing competing models, however, Section 2 of the appendix shows that all these performance measures rank models in the same way. There we also provide details on estimating $PB_{M}^{\left(0\right)}$ and $PB_{M}^{\left(1\right)}.$ In addition, in Section 3 of the appendix we show how to estimate the difference in *PB* between two completing models.

The definition of $PB_{M}^{\left(0\right)}$ is relevant to the net benefit described by Vickers et al.^29^ In more detail, net benefit (for a binary outcome) is defined as the reduction in event rate after following the model compared to the strategy “treat no-one” (ie, $PB_{M}^{\left(0\right)}$) minus the harms from treatment. Harms are quantified by Vickers et al. as the percentage of patients being treated following the model, multiplied by a value capturing the ratio of disutility of an event over that of treatment (eg, due to costs or side effects). Thus, net benefit is aimed at aiding decision making by providing a benefit vs harms assessment of a model. Conversely, we are here only interested in measures of predictive performance. We describe a range of alternative measures, that is, *PB*, $PB_{M}^{\left(0\right)},PB_{M}^{\left(1\right)}$ and we provide additional estimating procedures, that is, that account for imbalance in covariates. We also refer our readers to the penultimate paragraph of the Discussion.

Finally, note that the methods of this paragraph are somewhat similar to the approach used by Nguyen et al.,^30^ who proposed estimating treatment effect separately in patients with positive and negative values for the predicted treatment benefit ${\hat{B}}_{iM}.$

#### 4.4.2 Benefit accuracy: clustering using covariates

Another method to assess decision accuracy of a model is to estimate the proportion of patients in the population for whom the sign of *B*_*i*_ − *B*_*Th*_ matches that of ${\hat{B}}_{iM}-B_{Th}.$ The performance measure of interest, which we call benefit accuracy, is the following (assuming *B*_*Th*_ = 0 for simplicity):

(3) $$BA_{M}=P\left({\hat{B}}_{iM}>0\;\text{\&}\;B_{i}>0\right)+P\left({\hat{B}}_{iM}<0\;\text{\&}\;B_{i}<0\right),$$

where a perfect model will have *BA*_*M*_ = 100%.

To estimate ${\hat{\text{BA}}}_{M},$ we cluster “similar” patients as discussed in Section 4.3.2, and we estimate the predicted and observed benefit within each group. Then, we compare the sign of the observed with the sign of the predicted treatment benefit and count the proportion of groups where the two signs were concordant. We repeat the procedure multiple times and then average, to obtain stable results.

A problem with this approach is again that (unlike the method described in Section 4.4.1) the estimation needs to use covariates. It may thus be highly dependent on the set of covariates used to create the groups, as well as the number of groups. This may work well when, within the created groups the sign of ${\hat{B}}_{iM}$ and *B*_*i*_ remains relatively constant. Otherwise, this estimating procedure may fail. In that case, the estimates may be only useful for comparing models, rather than assessing their absolute performance. See the Discussion for more considerations on this point.

In Section 4 of the appendix, we describe an alternative estimation method, where instead of clustering patients, we match them one-on-one. All performance measures and estimators proposed in this article are summarized in Table 2.

## 5 Results

All methods described above are implemented in the R package predieval, freely available from https://github.com/esm-ispm-unibe-ch/predieval. In the appendix, we provide more details.

### 5.1 Analysis of the simulated dataset

We used the simulated dataset presented in Section 3.1, and we fitted the two predefined prediction models (*M*_1_ and *M*_2_). Then, we assessed the internal performance of the two models using all methods presented in this article. For this, we followed a 10-fold CV procedure repeated 100 times, to obtain out-of-sample predictions for all patients. Using these predictions, we estimated all measures. Since this was a simulated example, we could also calculate the true value of the performance measures. To this end, we generated a very large (external validation) dataset of 10 000 patients from the population, and we used the models developed in the original dataset to make predictions.

**Figure 2:**
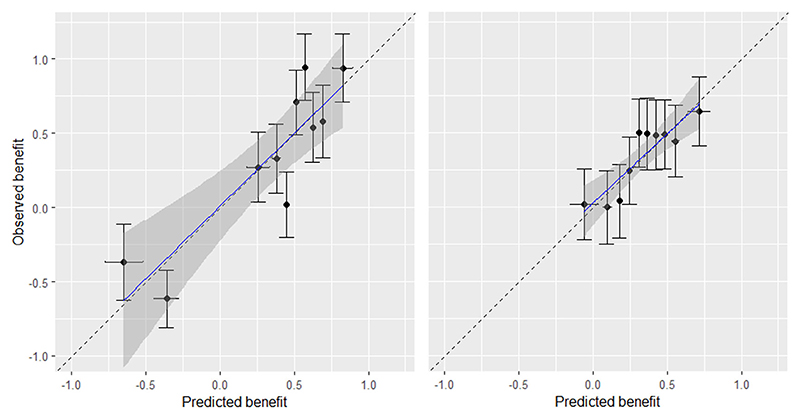
Calibration plot for treatment benefit predictions from models *M*_1_ (left) and *M*_2_ (right) in the simulated dataset with a continuous outcome. The plot was generated using the bencalibr *function of the* predieval *package in R*

We started our assessment with calibration for benefit. We found mean bias to be 0.04 for both *M*_1_ and *M*_2_. The true values of mean bias was −0.03 for both models. Next, we created a calibration-for-benefit plot after grouping patients into *N*_*g*_ = 10 groups according to ${\hat{B}}_{iM}$ (Section 4.3.1), shown in Figure 2. We saw that although both models were well calibrated, *M*_1_ performed better than *M*_2_, as it was able to capture more treatment effect heterogeneity. Then, we followed the methods of Sections 4.3.1 and 4.3.2, and did the analyses for *N*_*g*_ = 10, 20. Results are shown in Table 3, where we also provide the true values of the performance measures. Finally, we followed the method of Section 4.3.3 to fit a regression for benefit, and the slope for benefit was 0.97 [0.78; 1.15] for *M*_1_ vs 0.94 [0.65; 1.23] for *M*_2_.

Next, we turned to decision accuracy. For population benefit, we found ${\hat{PB}}_{M_{1}}=0.54[0.41;0.66],$ ${\hat{PB}}_{M_{2}}=0.34[0.21;0.47].$ These were estimated using the regression-adjustment method discussed in Section 4.4.1 Following the method described in Section 3 of the appendix (ie, unadjusted method), we estimated the difference between the two to be 0.18 [−0.03; 0.40], i.e. the method correctly suggested model *M*_1_ to be better, albeit with uncertainty. The true values (in the large, external population) were $PB_{M_{1}}=0.48$ and $PB_{M_{2}}=0.32.$ Next, we estimated benefit accuracy following the clustering method of Section 4.4.2, where we repeated 500 times, for *N*_*g*_ = 10, 20. Results for model *M*_1_ were 92% and 86%, and for *M*_2_ 91% and 80% for *N*_*g*_ = 10, 20, respectively, that is, correctly suggesting *M*_1_ to be better. The true values of the performance measures were 95% and 79% for *M*_1_ and *M*_2_, respectively.

We conclude that most evaluation methods correctly indicated that *M*_1_ yields superior estimates of individual treatment benefit, despite the fact that *M*_2_ was better in absolute outcome prediction. We also noted that in some instances, different estimation methods give very different results. We come back to this point in the Discussion section.

### 5.2 Analysis of the depression dataset

#### 5.2.1 Continuous outcome: symptoms severity in PHQ-9

The mean outcome after 9 weeks was 8.3 in PHQ-9 for the “switch to Mirtazapine” treatment arm (*t* = 1), 8.1 for the “combination” arm (*t* = 0), i.e. a clinically insignificant difference on average. For illustration purposes we explored three different modelling strategies to predict the outcome. These strategies were not tailored to identify treatment effect heterogeneity, but we used them for illustration purposes, to explore the performance of the proposed measures. The first strategy was to fit a linear regression model using all available predictors and all treatment-covariate interactions. The second was a ridge regression with the same structure as above but including penalization for all model parameters. We used a 10-fold CV to identify the optimal value for the tuning parameter of the model. The third strategy was to use a support vector machine (SVM) with a radial kernel for each treatment arm separately, using again an embedded 10-fold CV to select the tuning parameters. We fit the ridge using glmnet^31^ and the SVMs using the caret package in R.^32^ To assess model performance, we first obtained out-of-sample estimates via a 10-fold CV. We first compared the three strategies with respect to their performance in predicting the absolute outcome, across both arms. Results are shown in Table 4. We saw that ridge and SVM performed better than the unpenalized model in absolute outcome predictions.

We are also interested in identifying what treatment each patient should receive and predicting patient-level treatment benefit. This is potentially relevant, as all models predicted a wide range of patient-specific treatment benefit (ie, difference in PHQ-9 score), ranging from around −15 to +13 for the linear regression model and around −5 to +6 for the other two strategies. Such values are clinically meaningful and could be used to guide treatment decisions. We employed all methods described in this article; results are shown in Table 5.

**Figure 3:**
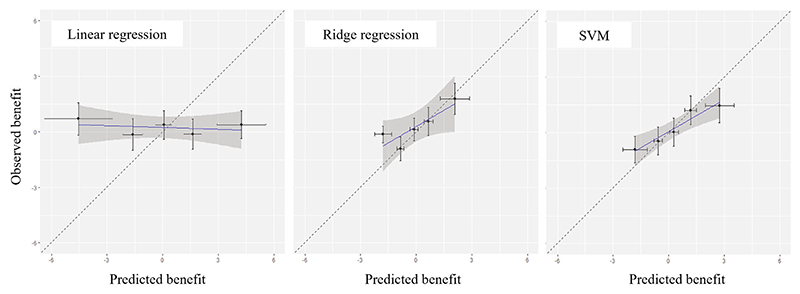
Calibration plots for benefit, for three competing models developed on the antidepressant example. The outcome is depression symptoms, measured on the PHQ-9 scale. SVM: support vector machines

First, in Figure 3 we show the calibration plots for benefit, for the three different strategies, and five groups. Linear regression seemed to perform worse, failing to find groups of patients that might benefit from treatment in different degrees. SVM performed slightly better than ridge, with the points in the graph being a bit closer to the diagonal. Next, we calculated all other measures for calibration for benefit described in this article, using 100 repetitions. In Table 5, we present results in terms of bias, RMSE, slope and *R*^2^. Results suggest again that the linear regression model performed worse than the other two models in all measures.

Then we turned to decision accuracy. We first estimated $\hat{PB},$ where ridge and then SVM performed again better. To allow us to gauge these results, note that the simplest prediction model, that is, one that would just predict the average outcome per treatment arm (ie, 8.3 and 8.1 respectively) would have $\hat{PB}=0.2.$ For estimating benefit accuracy, we used k-means clustering for *N*_*g*_ = 10 and 50; see Table 5. Results suggested again that SVM and then ridge performed better.

We conclude that ridge and SVMs seemed to perform similarly for this example, and that both were better than the unpenalized regression. All other things being equal, and following Occam’s razor (ie, among two similarly performing models, choose the simplest one), we would probably prefer ridge over the SVMs. After selecting ridge, to help better assess absolute model performance, we can also estimate *PB*^(0)^ and *PB*^(1)^. These were defined in Section 4.4.1, and we used formulas in the appendix to estimate standard errors. Results were $PB_{ridge\;}^{\left(0\right)}=0.5[0.0;1.0]$ and $PB_{ridge\;}^{\left(1\right)}=0.3[0.0;0.7],$ showing evidence that the use of this prediction model might provide a small benefit at the population level, as compared to just prescribing *t* = 0 or *t* = 1 to every patient. Moreover, we see at Figure 3 that the model may help identify group of patients for which the benefit is more pronounced (Table 4).

#### 5.2.2 Binary outcome: remission

A total of 343 patients out of 1032 (33%) remitted 6 weeks post randomization, 31% in the switching arm (*t* = 1) and 36% in the combination arm (*t* = 0). Aiming to illustrate all methods presented in this article, we evaluated two different modeling strategies. The first was an unpenalized logistic regression model with the following predictors: treatment, age, sex, years of education, and the nine items of PHQ-9 at baseline. We also included the interactions of all these covariates with treatment. The second modeling strategy was a stochastic gradient boosting machine (GBM), where tuning parameters were chosen after a 10-fold CV (embedded in the overall 10-fold CV), repeated 3 times. We fit a separate GBM in each treatment arm, using caret in R.^32^ To assess performance of these two strategies, we followed a 10-fold CV to obtain out-of-sample predictions for the risk for remission for each patient under each treatment. Using these, we assessed the performance of the models for predicting the outcome. There, we saw very similar results. The AUC was 0.77 and 0.76 for the logistic regression and GBMs respectively. Likewise, the mean predicted event rate in the two treatment arms was similar across models, very close to the true event rates (36% and 31%, respectively). Using the rms package in R^33^ we drew calibration plots^1^ (not shown here), and results were very similar: intercept −0.07 vs -0.06, slope 0.86 vs 0.90. These results suggest that there are no important differences in the performance of the models, and we could choose logistic regression because of its simplicity.

**Figure 4:**
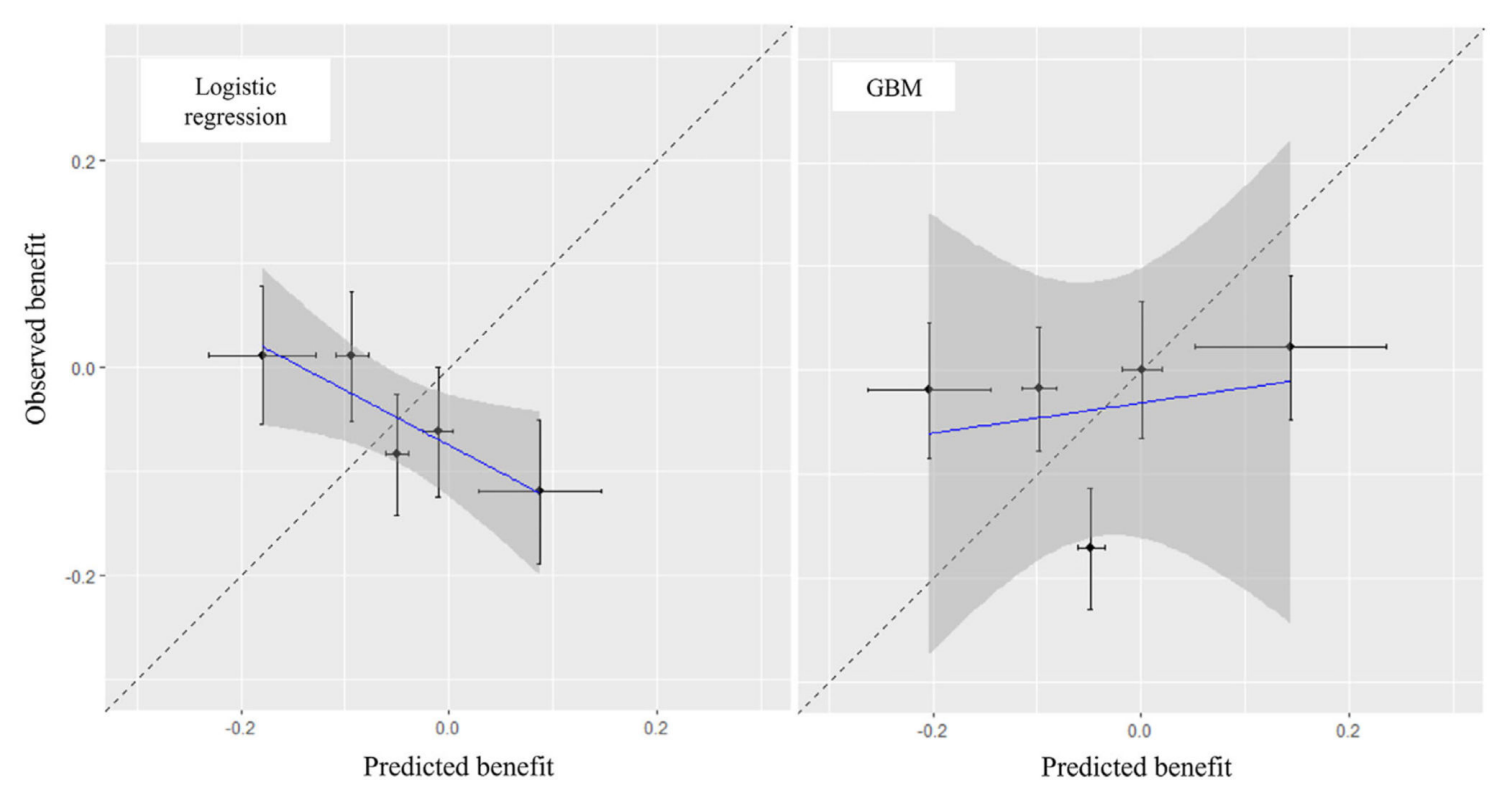
Calibration plots for benefit, for two competing models developed on the antidepressant example. The outcome is binary response. GBM: Gradient Boosting Machine

Next,we compare models in terms of treatment benefit. All results are given in Table 5. First, regarding discrimination for benefit,we sawthatGBMs performed very slightly better for C-for-benefit, but confidence intervals greatly overlapped. Next, we examined calibration for benefit. The calibration for benefit plot is shown in Figure 4, for *N*_*g*_ = 5, where we saw that the logistic regression model failed to predict treatment benefit across different groups.Mean biaswas almost zero for both methods. Results for all other measures of calibration for benefit are shown in Table 5, with the GBM outperforming logistic regression in all aspects. Interestingly, we see that the estimated slopes from logistic regression when we grouped by benefit were negative, reflecting the negative slope of the calibration plot in Figure 4. In terms of decision accuracy, we first assessed population benefit, where logistic regression also seemed to perform worse than GBM. Note that the simplest strategy to just predict for every patient the mean of the corresponding treatment group would give $\hat{PB}=4.7\text{\%}$ [0.0%; 10.4%] (ie, the risk difference between the two groups). Logistic regression ($\left(\hat{PB}=2.1\text{\%}\right)$), performed worse than even this simplest approach. Secondly, we saw that GBMs performed slightly better also for $\hat{BA}.$

Overall, we conclude that the strategy of using a GBM per treatment arm clearly outperformed the single logistic regression model, when it comes to treatment benefit, although they had very similar performance in absolute outcome prediction.

Finally, to assess the absolute performance of the selected model (GBM), we can also estimate *PB*^(0)^ and *PB*^(1)^. We found ${\hat{PB}}_{GBM}^{\left(0\right)}=0.5\text{\%}[-3.0\text{\%};3.9\text{\%}]$ and ${\hat{PB}}_{GBM}^{\left(1\right)}=5.2\text{\%}[0.6\text{\%};9.7\text{\%}].$ Essentially, we found almost no evidence that treating patients according to the GBM model would lead to better population results than just using the simplest strategy of giving everyone *t* = 0. Figure 4 also suggests that the model offers little in terms of identifying patients that might benefit more/less from treatment. We conclude that for the analysis of the binary outcome, our two prediction models failed to identify any meaningful treatment effect modification. Thus, we saw that even though the sample size in this example was big (1032 patients), the uncertainty associated with estimating treatment benefit at the patient level was so large that precluded any firm conclusions Table 5.

## 6 Discussion

Prognostic models typically aim to predict the absolute risk of future outcomes, for example, mortality risk 6 months after diagnosis. When such models are used to inform treatment decisions, it is important to assess their ability to accurately predict treatment benefit, for example, the reduction or increase in the risk of an event for a patient, when receiving treatment. We hereby started by defining two dimensions of accuracy when predicting treatment benefit, that is, discrimination for benefit and calibration for benefit, by extending definitions used from prognostic modelling.^1^ We also defined the decision accuracy of a model, which combined calibration and discrimination for benefit. All these aspects of accuracy should ideally be assessed when selecting among competing modeling strategies for assessing treatment benefit. Their relative importance may be context-specific, that is, it may depend on the decision-making framework in which the prediction model will be embedded. For instance, when the outcome is death, and when costs of intervention are of no concern, decision accuracy is probably of primary importance. In this situation we want a model that will best identify patients who should receive treatment (ie, patients with positive treatment benefit) and patients who should not (patients with negative treatment benefit), as this would prevent unnecessary deaths. Conversely, if decision-making is based on information about multiple efficacy and safety outcomes, calibration measures may be more important, particularly if costs are also an issue and when predicted treatment effects are to be included in a health economic model. For instance, when treatment is associated with side effects or when it is expensive, we might want to prioritize treating patients for whom the benefit from receiving treatment is expected to be large.

Next, we discussed a series of performance measures related to these aspects of accuracy for treatment benefit. For some of these measures we proposed alternative estimating procedures, for example, grouping patients according to their characteristics or according to predicted benefit. The results of the simulated dataset showed that our metrics have the potential to identify models that provide better estimates of treatment benefit. At the same time, both the results from the simulated dataset and from the real medical example showed that different estimators may give different estimates, that is, estimates may greatly depend on the estimating procedure. Of note, some of the estimating procedures are based on strong assumptions, some of which may not hold in real applications. For example, when we cluster using covariates, we try to create groups of “similar” patients, and we assume that within each cluster the true benefit is relatively constant across all patients. In the appendix, we provide an additional estimating method for some of the metrics, based on matching patients one-on-one. Matching was also used in a recent article by Maas et al., which described methods for estimating performance measures for individualized treatment effects.^19^ Matching, however, rests on very strong assumptions (ie, that the baseline risk is the same in each matched pair), so we expect it to be suboptimal. Thus, for some of the metrics we proposed, the estimated values may not be good approximations of the true values. In that case, these estimates may be better thought of as “statistics”, i.e. metrics to be compared across models rather than measures of absolute model performance. In any case, a systematic simulation study is needed to help decide which estimators are most efficient and least biased, that is, which ones can be used for model evaluation and which ones for model comparison; we leave this for future work. Related to that, it would also be interesting to compare in simulations our measures with alternative methods.^18,19,25^ Moreover, we only discussed a single method for discrimination for benefit, the C-statistic proposed by van Klaveren et al.^24^ Although originally proposed for binary outcomes, using the method for continuous outcomes is straightforward: we would first match patients on treatment with patients on control (according to their covariates or the predicted benefit); then, measure the probability that from two randomly chosen matched pairs, the pair with greater observed benefit also has a higher predicted benefit. However, we did not further pursue this idea, given the possible limitations of this method.^9^

Of note, when setting up our framework, for binary outcomes we assumed stochastic events, where the treatment benefit is defined to be the difference in the probability of an event in treatment minus that in control. Instead, we could have assumed deterministic counterfactuals, where the latter can be technically thought of as a special case of the former (ie, a deterministic counterfactual is the limit of a stochastic one, when the corresponding probability is 0 or 1). In that case, the “true benefit” could only take three values, that is, −1, 0, and 1 (while in this article we assumed it is continuous, on the [−1,1] interval). Apart from this conceptual change, methods described in this article would not be affected. However, we think there is no a priori reason why the latter choice is preferable to the former. Moreover, on a practical level, simulations are simpler to perform when we assume stochasticity of the events. Thus, in this article, we assumed stochastic events (instead of fixed counterfactuals), as they facilitate the developing and testing of the methods presented in this article.

In all our analyses, we assumed fully observed data. In practice, for some of the participants, we may have missing predictors. In such cases, we can first use a multiple imputation approach to impute the missing data,^34^ and then follow all procedures described in this article (including obtaining out-of-sample predictions), and summarize all performance measures from the imputed datasets at the end, using Rubin’s rules.^35^ Moreover, the analyses presented in this article used randomized data. When the main interest is in treatment effects, randomized clinical data generally represent the best source of information because they do not require adjustment for confounding. However, all methods could in principle also be extended for models developed on observational data (with some refining, for example, in the assessment of mean bias). In such cases, the usual assumptions of causal inference from observational studies need to hold, that is, proper adjustments for confounding, measurement bias, selection bias, etc.^36^

Another important limitation is that, irrespective of the choice of performance measure or estimators, all methods presented in this article are expected to have limited power to find differences between competing models. Estimating treatment effects in different patient groups is challenging to begin with, and, depending on the setting, it may require large amounts of data.^37^ At the same time, RCTs are usually only powered to detect treatment effects at the population level.^14,38^ Moreover, comparing slightly different models for treatment benefit will be even more challenging in most cases, unless the dataset is extensive. This issue was highlighted in the analyses described in this article, both the ones using simulated and the ones using real data. In these analyses we saw that the assessment of treatment benefit was associated with large uncertainty, even though the sample sizes we used would probably be considered large in practical applications. This was especially problematic for the case of binary outcomes (and serves therefore as another argument to why dichotomization should be avoided). Thus, in general, we recommend that researchers should be particularly cautious when trying to explore effect modification in usual cases of data availability.

For these reasons, extending our approaches to use in large observational datasets may be particularly useful. An IPD meta-analysis of many RCTs^39^ offers another way to increase sample size, by putting together data from multiple studies. The use of data from multiple sources bears the extra advantage of better allowing us to evaluate the generalizability of predictions to new settings.^40^ The problem is that obtaining IPD from multiple studies is often difficult in practice, while IPD can be prone to substantial between-study heterogeneity, for example, when studies adopt different variable definitions, measurement methods and other design choices. Initiatives such as YODA (https://yoda.yale.edu/) and Clinical Study Data Request (https://www.clinicalstudydatarequest.com/) aim to promote the sharing of IPD, hopefully facilitating the conduct of more IPD-NMAs in the future.^41^ Our methods would require adjustments to use in a meta-analytical setting; this is also an area of interesting future research. Moreover, for most clinical conditions there are multiple competing interventions to choose from. Thus, it would be interesting to extend all methods presented for comparing more than two treatments (as was the case in the depression dataset presented in this article), and eventually to an IPD network meta-analysis setting.^42,43^

Finally, when developing our methods, we assumed a common treatment threshold *B*_*Th*_ for all patients. However, we could think of cases when this value is different for different types of patients, for example, when some patients are at a higher risk of an adverse effect. In that case the threshold for treatment might be higher. In such cases, if we can write *B*_*Th*_ as a function of patient covariates, we can easily change the definitions of our measures in Section 4.4 to accommodate variability in thresholds. Then, the treatment recommendation for patient *i* would be decided after comparing ${\hat{B}}_{iM}$ to *B*_*i,Th*_.

Generally, however, we acknowledge that personalizing the decision-making process is a very complicated procedure in practice, as it should consider many aspects, such as patient preferences, likelihood of multiple side effects, multiple efficacy outcomes, costs, etc. A cost-benefit analysis,^29^ a decision curve analysis,^44,45^ or a multiple criteria decision analysis (MCDA^46^) can all inform health-care decisions and facilitate a shared decision making between patients and doctors. MCDA aims to do so by balancing costs, benefits and risks of the interventions, while also taking into account the personal preferences of the patients. A range of models that predict treatment benefits and harms (ie, treatment effects for multiple effectiveness and safety outcomes) would be needed to inform such an analysis. The current article only considered the issue of model evaluation and model selection for a single outcome, which might be a very small part of this bigger process. However, we still think it is important to make best use of existing data when developing a new prediction model for benefit, as it may be used to guide treatment decisions.

To summarize, in this article we proposed a range of measures for assessing the performance of models aiming to predict patient-level relative effects among two interventions, and we provided freely available software which can used to improve our understanding of these methods as well as facilitate their uptake in practice.

## Supplementary Material

Appendix S1: Supporting Information

## Figures and Tables

**Table 1:** A list of notation used in this article

Notation	Description
*i*	Patient indicator
***x***_***i***_ = (*x*_*i*1_, *x*_*i*2_ …)	A set of patient-level covariates for patient *i*.
*M*	A model that uses covariates ***x*** to predict outcomes under treatment and control.
*t_i_*	Treatment indicator: 1 for treatment, 0 for control.
*y_t_*	Observed outcome. Can be either continuous or binary.
*ŷ*_*iM,t*=1_	Patient-level prediction of a continuous outcome under active treatment, as obtained from model *M*. This is on the scale of the original outcome.
*ŷ*_*iM,t*=0_	Patient-level prediction of a continuous outcome under control, as obtained from model *M*. This is on the scale of the original outcome.
${\hat{p}}_{iM,t=1}$	Patient-level predicted probability of an event for a binary outcome under treatment, as obtained from model *M*.
${\hat{p}}_{iM,t=0}$	Patient-level predicted probability of an event for a binary outcome under control, as obtained from model *M*.
*B_i_*	True underlying patient-level benefit. This is unobservable.
*B_Th_*	A clinically meaningful threshold for the treatment benefit.
${\hat{B}}_{iM}$	Patient-level benefit estimated from model M. It is equal to *ŷ*_*iM,t*=1_ − *ŷ*_*iM,t*=0_ for continuous outcomes, ${\hat{p}}_{iM,t=1}-{\hat{p}}_{iM,t=0}$ for binary outcomes.
$PB_{M},PB_{M}^{\left(0\right)},\;\text{and}\;PB_{M}^{\left(1\right)}$	Measures of population-level benefit after using model *M* to make treatment decisions. Defined in Section 4.4.1
*BA*_*M*_	Benefit accuracy: proportion of patients in the population for whom the sign of *B*_*i*_ matches that of ${\hat{B}}_{iM}.$ Defined in Equation (3).
bias, RMSE	Bias and root mean squared error when comparing two sets of values.
*a*_0_, *a*_1_, *R*^2^	Intercept, slope, and coefficient of determination obtained after fitting a linear regression on two sets of values.

**Table 2:** Summary of the proposed dimensions for measuring model accuracy with respect to treatment benefit, performance measures, and estimating procedures

Dimension	Performance measure	Estimating procedures	Section
*Discrimination for benefit* The ability of a model to differentiate patients who will benefit more from patients who will benefit less from treatment	C-for-benefit (binary outcomes only)	Matching algorithm by van Klaveren et al.^24^ Matching can use covariates or ${\hat{B}}_{iM}.$	4.2
*Calibration for benefit* The ability of a model to estimate the magnitude of treatment effects.	Mean bias: mean difference between true and predicted treatment benefit $E\left(B_{l}-{\hat{B}}_{lM}\right)$	Compare the difference of mean outcomes among treatment arms with the mean ${\hat{B}}_{iM}$	4.3.1
	Intercept, slope and *R*^2^ of the $B_{i}\sim {\hat{B}}_{iM}$ line Root mean squared error (RMSE): $\sqrt{E\left({\left(B_{1}-{\hat{B}}_{iM}\right)}^{2}\right)}$	Group according to ${\hat{B}}_{iM}$ and regress mean observed on mean-predicted treatment effect	4.3.1
		Cluster patients using covariates and regress mean observed on mean-predicted treatment effect	4.3.2
		Regress observed outcome on predicted outcome in control and on treatment-benefit interaction	4.3.3
		“Matching patients one-to-one” algorithm. Matching can be repeated multiple times, using covariates or ${\hat{B}}_{iM}.$	Appendix Section 4.2
*Decision accuracy* The ability of a model to identify patients who would benefit by at least *B*_*Th*_, from receiving treatment (*t* = 1) rather than control (*t* = 0).	*PB*_*M*_, population-level benefit of following model *M* vs following the opposite of *M*. Equation (1)	Equation (2)	4.4.1
		Covariate-adjusted version of Equation (2), using regression.	4.4.1
		Covariate-adjusted version of Equation (2) using inverse probability weighting.	4.4.1
	$PB_{M}^{\left(0\right)}$ and $PB_{M}^{\left(1\right)},$ population-level benefit of following model *M* vs treating everyone with *t* = 0 or *t* = 1 *BA*_*M*_, benefit accuracy: proportion of patients in the population for whom the sign of *B*_*i*_ − *B*_*Th*_, matches that of ${\hat{B}}_{iM}$ − *B*_*Th*_. Equation (3)	Appendix Section 2	
		“Clustering using covariates” algorithm	4.4.2
		“Matching patients one-to-one” algorithm. Matching can be repeated multiple times, using covariates or ${\hat{B}}_{iM}.$	Appendix Section 4.1

**Table 3:** Calibration for benefit from the simulated dataset with continuous outcome, comparing models M_1_ and M_2_

Performance measure estimation method	Model *M*_1_					Model *M*_2_			
	RMSE	*a*_0_	*a*_1_	*R* ^2^		RMSE	*a*_0_	*a*_1_	*R* ^2^
True values of the performance measure, estimated using 10 000 new patients	0.12	−0.05	1.08	0.95		0.41	−0.11	1.25	0.32
Group by benefit, *N_g_ =* 10	0.23	0.01	0.98	0.78		0.10	0.03	0.93	0.79
Group by benefit, *N_g_* = 20	0.31	0.01	0.98	0.67		0.23	0.03	0.87	0.42
k-means, *N_g_ =* 10	0.16	−0.07	1.29	0.69		0.16	−0.04	1.19	0.67
k-means, *N_g_* = 20	0.23	−0.09	1.34	0.71		0.36	−0.11	1.16	0.33

*Note*: Estimates obtained following a 10-fold CV repeated 100 times. M_2_ performed better in predicting the absolute prognostic outcome (results given in text), but this table shows that it performed worse in predicting treatment benefit.

**Table 4:** Comparing three modeling strategies (linear regression, ridge regression, and support vector machines [SVMs]) for continuous outcome prediction in the antidepressants dataset

Measure of performance		Unpenalized linear regression	Linear regression with ridge penalty	One SVM per treatment arm
*Comparing predicted vs observed outcomes*				
Bias		0.0	0.0	0.3
RMSE		5.1	4.5	4.5
*R* ^2^		0.32	0.42	0.43
*Calibration for benefit*				
Mean bias for benefit		0.20	0.16	−0.21
Group by benefit, *N_g_ =* 10	RMSE	3.5	0.9	1.2
	slope	−0.10	0.55	0.63
	*R* ^2^	0.14	0.56	0.48
Group by benefit, *N_g_ =* 50	RMSE	4.1	2.2	2.6
	slope	−0.08	0.62	0.66
	*R* ^2^	0.01	0.12	0.15
Group by k-means, *N_g_ =* 10	RMSE	2.3	1.3	1.0
	slope	−0.95	0.67	1.16
	*R* ^2^	0.54	0.43	0.63
Group by k-means, *N_g_ =* 50	RMSE	2.9	2.0	1.9
	slope	−0.87	0.68	1.19
	*R* ^2^	0.28	0.24	0.43
Regression for benefit slope		0.28 [0.14; 0.42]	0.53 [0.16; 0.90]	0.76 [0.52; 1.00]
*Decision accuracy*				
$\hat{PB}$		0.4 [−0.2; 0.9]	0.6 [0.0; 1.2]	0.5 [−0.1; 1.0]
$\hat{BA},$ k-means, *N_g_* = 10		29%	67%	68%
$\hat{BA},$ k-means, *N_g_ =* 50		38%	65%	68%

*Note*: Brackets denote 95% Confidence intervals.

Abbreviations: BA, benefit accuracy; MSE, mean squared error; PB, population benefit.

**Table 5:** Comparing two modeling strategies for predicting binary response in the antidepressants dataset

Measure of performance		Logistic regression	GBM
*Comparing predicted vs observed outcomes*			
AUC		0.77	0.76
Calibration plot intercept		−0.07	*−*0.06
Calibration plot slope		0.86	0.90
*Discrimination for benefit*			
c-for-benefit, match by covariates		0.51 [0.47; 0.56]	0.53 [0.48; 0.58]
c-for-benefit, match by benefit		0.50 [0.46; 0.55]	0.50 [0.46; 0.55]
*Calibration for benefit*			
Mean bias for benefit		0.0%	0.2%
Group by benefit, *N_g_ =* 5	RMSE	0.13	0.12
	slope	*−*0.52	0.15
	*R* ^2^	0.80	0.06
Group by benefit, *N_g_ =* 10	RMSE	0.15	0.13
	slope	*−*0.42	0.23
	*R* ^2^	0.22	0.09
Group by k-means, *N_g_ =* 5	RMSE	0.04	0.04
	slope	1.87	1.19
	*R* ^2^	0.55	0.46
Group by k-means, *N_g_ =* 10	RMSE	0.05	0.05
	slope	1.87	1.19
	*R* ^2^	0.55	0.46
Regression for benefit slope		0.40 [0.07; 0.75]	0.60 [0.30; 0.91]
*Decision accuracy*			
$\hat{PB}$		2.1% [−2.9%; 7.2%]	6.3% [1.2%; 11.2%]
$\hat{BA},$ k-means, *N_g_* = 5		80%	79%
$\hat{BA},$ k-means, *N_g_* = 10		82%	84%

Abbreviations: GBM, gradient boosting machine; MSE, mean squared error.

## Data Availability

The data cannot be made publicly available due to confidentiality agreements.
