# Supplementary material for: Measuring the performance of prediction models to personalize treatment choice
Source: Stat Med. Author manuscript; Available in PMC 2024 Mar 8. (PMC7615726; doi:10.1002/sim.9665)
Supplement: Appendix S1: Supporting Information [file EMS194374-supplement-Appendix_S1__Supporting_Information.docx]

**Appendix**

**Measuring the performance of prediction models to personalize treatment choice**

*Orestis Efthimiou^1,2,3 *^, Jeroen Hoogland^4^, Thomas P.A. Debray^4,5^, Michael Seo^1,6^, Toshiaki A. Furukawa^7^, Matthias Egger^1^, Ian R. White^8^*

*^1^ Institute of Social and Preventive Medicine (ISPM), University of Bern, Bern, Switzerland*

*^2^ Institute of Primary Health Care (BIHAM), University of Bern, Bern, Switzerland*

*^3^Department of Psychiatry, University of Oxford, Oxford, United Kingdom*

*^4^ Julius Center for Health Sciences and Primary Care, University Medical Center Utrecht, Utrecht University, Utrecht, The Netherlands*

*^5^ Smart Data Analysis and Statistics B.V., Utrecht, The Netherlands*

*^6^Graduate School for Health Sciences, University of Bern, Bern, Switzerland*

*^7^Departments of Health Promotion and Human Behavior and of Clinical Epidemiology, Kyoto University Graduate School of Medicine/School of Public Health, Kyoto, Japan*

*^8^ MRC Clinical Trials Unit at UCL, University College London, London, UK*

** corresponding author, email: oremiou@gmail.com*

Table of Contents

[**1** **Internal validation of prediction models** 2](#_Toc115702712)

[**2** **Notes on the measures for population benefit** 2](#_Toc115702713)

[***2.1*** ***Relationship between different performance measure for population benefit*** 2](#_Toc115702714)

[***2.2*** ***Estimation procedures*** 3](#_Toc115702715)

[***2.3*** ***Estimating the difference in population benefit between different models*** 4](#_Toc115702716)

[**3** **Estimation via matching patients one-on-one** 6](#_Toc115702717)

[***3.1*** ***Decision accuracy via matching*** 6](#_Toc115702718)

[***3.2*** ***Calibration for benefit via matching (continuous outcomes only)*** 6](#_Toc115702719)

[**4** **Details about the toy dataset shown in the main paper** 7](#_Toc115702720)

[***4.1*** ***Details about data generation*** 7](#_Toc115702721)

[***4.2*** ***Results after matching*** 8](#_Toc115702722)

[**5** **A simulated dataset with a binary outcome** 9](#_Toc115702723)

[***5.1*** ***Data generation*** 9](#_Toc115702724)

[***5.2*** ***Model development*** 10](#_Toc115702725)

[***5.3*** ***Results from the simulated dataset for binary outcomes*** 10](#_Toc115702726)

[**6** **Examples for using the predieval package in R** 12](#_Toc115702727)

[***6.1*** ***Continuous outcome*** 12](#_Toc115702728)

[***6.2*** ***Binary outcome*** 14](#_Toc115702729)

[**7** **References** 17](#_Toc115702730)

# **Internal validation of prediction models**

It is recommended to assess the performance of a prediction model in new, external data that was not used for model development. Ideally, this would be performed using data from a new RCT, where the relevant models can be applied. Unfortunately, external validation is not always possible, as it is often hard to obtain data from multiple trials. It is therefore desirable to assess and compare model performance in the development data. However, if performance is assessed on the same data that we used to develop the model, we run the risk of overfitting and optimism, especially when the model is complex and/or the dataset is small. Overfitting means that the model fits the data very well but fails to predict accurately for new data. Optimism means that assessments of model performance are upwardly biased. Thus, we want a patient’s outcome to be predicted after training the model in data that did not include this patient. One way to do this is via data splitting (e.g. use 70% of the data for training and 30% for testing). A less wasteful approach is via k-fold cross-validation (CV). Briefly, this procedure involves the following steps

1. We predefine the different modelling strategies that we are interested in evaluating.
2. We randomly split the data into$k$ approximately equally sized folds. We remove one fold and use the remaining $k-1$folds to develop each of the competing models. We then use the covariates of the patients in the left-out fold to make predictions.
3. We cycle through all folds, each time leaving another fold out. We thus obtain predictions for each patient, for each treatment and from each modelling strategy.
4. We use performance measures (defined below) to evaluate model performance – and if model selection is of interest, to select the best performing model, $M_{*}$.
5. We refit model $M_{*}$ to the whole dataset to obtain the final model.

Note that, ideally, we should repeat the $k$-fold CV multiple times to obtain stable results. Alternatively, we could use a bootstrapping procedure^1^, which has been recommended for assessing the internal validity of a predictive model.^2^ Also note that for steps *ii* and *v* we may use another cross-validation procedure to develop the model, embedded within the overall k-fold CV. For example, in step *i* we may decide to fit a LASSO model, where the tuning parameter will be identified via 10-fold cross-validation, nested within the outer $k$-fold CV. Finally, note that this strategy does not protect 100% against overfitting. If we include in the evaluation a large number of models or if we start making data-dependent choices to maximize performance in step iv, we again run the risk of overfitting when evaluating the performance of the ‘best’ model. Thus, this approach is most protective against overfitting when a small number of predefined modelling strategies are compared.

# **Notes on the measures for population benefit**

## ***Relationship between different performance measure for population benefit***

Here we clarify the relationship between the performance measures discussed in Section 8.1 of the main paper. We start by noting that by definition

$$PB_{M}=E\left( y | treat according to model M_{X} \right)-E(y|treat with the opposite of model M_{X})$$

Also we have introduced the following performance measure in the paper

$$PB_{M}^{(0)}=E\left( y | treat according to model M_{X} \right)-E(y|t=0)$$

We will show that $PB_{M}$ and $PB_{M}^{(0)}$ are equivalent for model comparison. The key observation is that

$$E\left( y | treat according to model M_{X} \right)+E\left( y | treat with the opposite of model M_{X} \right)=E\left( y | t=0 \right)+E\left( y | t=1 \right).$$

This equation holds because if we follow $M_{X}$, each patient either receives $t=0$ or $t=1$. This means that in the left part of the previous equation we sum through the expected outcomes of all patients twice, once for $t=0$ and once for $t=1$, as shown in the right part. Next, the previous equations imply that:

$$PB_{M}=E\left( y | treat according to model M_{X} \right)-E\left( y | treat with the opposite of model M_{X} \right)= 2 E\left( y | treat according to model M_{X} \right)-E\left( y | t=0 \right)-E\left( y | t=1 \right)=$$

$$2 \left( E\left( y | treat according to model M_{X} \right)-E\left( y | t=0 \right) \right)+E\left( y | t=0 \right)-E\left( y | t=1 \right)=$$

$$2 PB_{M}^{(0)}+c$$

where $c=E\left( y | t=0 \right)-E\left( y | t=1 \right)$, i.e. a quantity not dependent on the model. Thus, when comparing two different models we can use either $PB_{M}$ or $PB_{M}^{(0)}$, as the one is just a linear transformation of the other. Likewise, the following performance measure is also equivalent when it comes to comparing models:

$PB_{M}^{(1)}=E\left( y | treat according to model M_{X} \right)-E(y|t=1)$.

## ***Estimation procedures***

For estimation of $PB_{M}$, the simplest method is to use the definition

$$\hat{PB}_{M}=\bar{y}_{G_{1}UG_{4}}-\bar{y}_{G_{2}UG_{3}}=\frac{\sum_{G_{1}} y_{i}+\sum_{G_{4}} y_{i}}{n_{1}+n_{4}}-\frac{\sum_{G_{2}} y_{i}+\sum_{G_{3}} y_{i}}{n_{2}+n_{3}}$$

Where $\sum_{G_{1}} y_{i}$ is used to denote $\sum_{i\in G_{1}} y_{i}$, i.e. the sum of outcomes in the $G_{1}UG_{4}$ group, and $\bar{y}_{G_{1}UG_{4}}$ denotes the mean outcome in the $G_{1}UG_{4}$ group. The corresponding variance

$$Var\left( \hat{PB}_{M}^{\left( 0 \right)} \right)=\frac{n_{1}Var\left( y_{i} | G_{1} \right)+n_{4}Var\left( y_{i} | G_{4} \right)}{\left( n_{1}+n_{4} \right)^{2}}+\frac{n_{2}Var\left( y_{i} | G_{2} \right)+n_{3}Var\left( y_{i} | G_{3} \right)}{\left( n_{2}+n_{3} \right)^{2}}$$

In the paper we describe two additional methods for estimating, that also adjust for covariates.

For the estimation of $PB_{M}^{(0)}$ we note that

$$\hat{PB}_{M}^{(0)}=\bar{y}_{G_{1}UG_{4}}-\bar{y}_{G_{2}UG_{4}}=\frac{\sum_{G_{1}} y_{i}+\sum_{G_{4}} y_{i}}{n_{1}+n_{4}}-\frac{\sum_{G_{2}} y_{i}+\sum_{G_{4}} y_{i}}{n_{2}+n_{4}}=$$

$$\frac{\sum_{G_{1}} y_{i}}{n_{1}+n_{4}}-\frac{\sum_{G_{2}} y_{i}}{n_{2}+n_{4}}+\sum_{G_{4}} y_{i}\left( \frac{1}{n_{1}+n_{4}}-\frac{1}{n_{2}+n_{4}} \right)$$

With a corresponding variance

$$Var\left( \hat{PB}_{M}^{\left( 0 \right)} \right)=\frac{n_{1}Var\left( y_{i} | G_{1} \right)}{\left( n_{1}+n_{4} \right)^{2}}+\frac{n_{2}Var\left( y_{i} | G_{2} \right)}{\left( n_{2}+n_{4} \right)^{2}}+n_{4}Var\left( y_{i} | G_{4} \right)\left( \frac{1}{n_{1}+n_{4}}-\frac{1}{n_{2}+n_{4}} \right)^{2}$$

Where $Var\left( y_{i} | G_{1} \right)$ denotes $Var\left( y_{i} | {i\in G}_{1} \right)$, i.e. the variance of the outcome at group $G_{1}$.

Likewise for $PB_{M}^{(1)}$:

$$\hat{PB}_{M}^{(1)}=\bar{y}_{G_{1}UG_{4}}-\bar{y}_{G_{1}UG_{3}}=\frac{\sum_{G_{1}} y_{i}+\sum_{G_{4}} y_{i}}{n_{1}+n_{4}}-\frac{\sum_{G_{1}} y_{i}+\sum_{G_{3}} y_{i}}{n_{1}+n_{3}}=$$

$$\frac{\sum_{G_{4}} y_{i}}{n_{1}+n_{4}}-\frac{\sum_{G_{3}} y_{i}}{n_{1}+n_{3}}+\sum_{G_{1}} y_{i}\left( \frac{1}{n_{1}+n_{4}}-\frac{1}{n_{1}+n_{3}} \right)$$

With a corresponding variance

$$Var\left( \hat{PB}_{M}^{\left( 1 \right)} \right)=\frac{n_{4}Var\left( y_{i} | G_{4} \right)}{\left( n_{1}+n_{4} \right)^{2}}+\frac{n_{3}Var\left( y_{i} | G_{3} \right)}{\left( n_{1}+n_{3} \right)^{2}}+n_{1}Var\left( y_{i} | G_{1} \right)\left( \frac{1}{n_{1}+n_{4}}-\frac{1}{n_{1}+n_{3}} \right)^{2}$$

## ***Estimating the difference in population benefit between different models***

Assume we have two models $M$ predicting treatment benefit with respect to a continuous outcome. As shown in Section 8.1 of the paper we can classify patients in four groups according to the predicted benefit from model $M$:


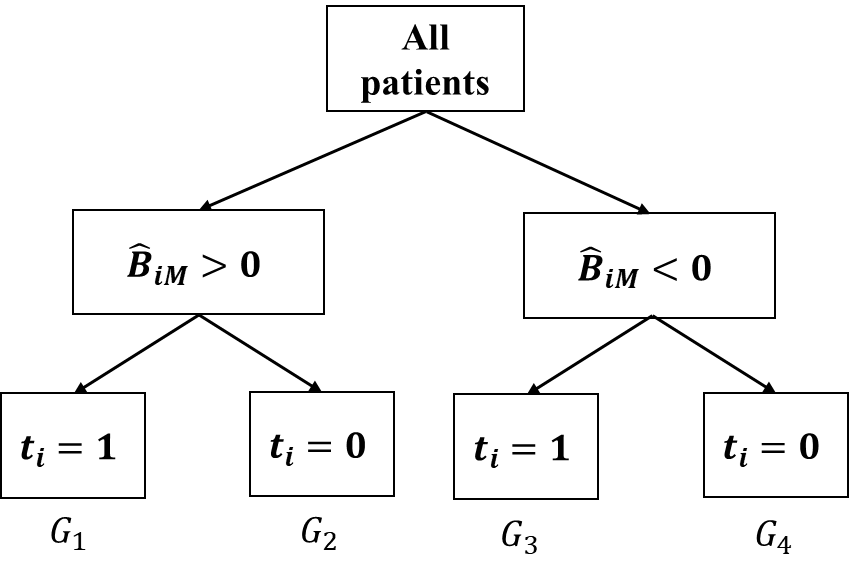


and then, as discussed above, we can estimate

$$\hat{PB}_{M}=\bar{y}_{G_{1}UG_{4}}-\bar{y}_{G_{2}UG_{3}}=\frac{\sum_{G_{1}} y_{i}+\sum_{G_{4}} y_{i}}{n_{1}+n_{4}}-\frac{\sum_{G_{2}} y_{i}+\sum_{G_{3}} y_{i}}{n_{2}+n_{3}}$$

In the paper we also discuss covariate-adjusted estimators, but here we only look into the unadjusted case.

Let us now assume we also have a different model, $M^{*}$. We can use the same procedure for $M^{*}$, to split patients in groups $G_{1}^{*}, G_{2}^{*}, G_{3}^{*}, G_{4}^{*}$ with sizes $n_{1*}, n_{2*},n_{3*}, n_{4*}$and estimate $\hat{PB}_{M^{*}}$. The question is then how to estimate ${\Delta\hat{PB}}_{M}=\hat{PB}_{M}-\hat{PB}_{M^{*}}$, and more specifically, the standard error of this quantity. This is non-trivial, given that the two quantities are estimated on the same patients, and this induces correlations that need to be accounted for. In what follows we will rearrange the terms in this equation, so that it can be estimated using four different, non-overlapping groups.

We start by the following definitions:

- Group $G_{14}=G_{1}\cup G_{4}$
- $n_{14}=n_{1}+n_{4}$
- $n_{23}=n_{2}+n_{3}$
- Group $G_{14}^{*}=G_{1}^{*}\cup G_{4}^{*}$
- $n_{14*}=n_{1*}+n_{4*}$
- $n_{23*}=n_{2*}+n_{3*}$

Obviously, $G_{14}$ and $G_{14}^{*}$ are overlapping. Then, we define the following new variables in our dataset (suppressing the patient index for simplicity):

- $g_{14}$ is a dummy indicator that a patient is included in group $G_{14}$ (i.e. received optimal treatment according to $M$)
- $g_{14*}$ is a dummy indicator that a patient is included in group $G_{14}^{*}$ (i.e. received optimal treatment according to $M^{*}$)
- $same$ is a dummy indicator that the two models agree that the patient received the optimal treatment, $g_{14}=g_{14*}$

The patients are now split in 4 new groups A, B, C and D, according to whether the two models agree on if a patient received the optimal treatment


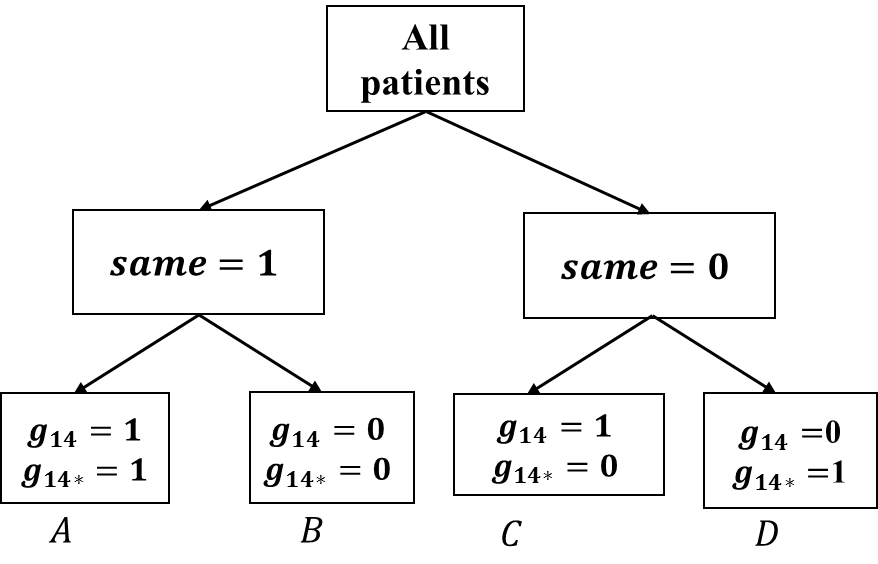


Group A includes all patients for whom $M$ and $M^{*}$ agreed on what is the optimal treatment, and the patient actually received this treatment.

Group B includes all patients for whom $M$ and $M^{*}$ agreed on what is the optimal treatment, but the patient actually did not receive this treatment.

Group C includes all patients for whom $M$ and $M^{*}$ did not agree on what is the optimal treatment, and the patient actually received the treatment predicted by $M$to be optimal.

Group D includes all patients for whom $M$ and $M^{*}$ did not agree on what is the optimal treatment, and the patient actually received the treatment predicted by $M^{*}$to be optimal.

We now note that

$${\Delta\hat{PB}}_{M}=\hat{PB}_{M}-\hat{PB}_{M^{*}}=\frac{\Sigma\left( y | g_{14}=1 \right)}{n_{14}}-\frac{\Sigma\left( y | g_{14}=0 \right)}{n_{23}}-\frac{\Sigma\left( y | g_{14*}=1 \right)}{n_{14*}}+\frac{\Sigma\left( y | g_{14*}=0 \right)}{n_{23*}}$$

where we suppressed the patient indicator on $y$ and $g$’s for simplicity. This expression can be further analysed, where each term above corresponds to one line below:

$${\Delta\hat{PB}}_{M}=$$

$$\frac{1}{n_{14}}\sum y g_{14} same+\frac{1}{n_{14}}\sum y g_{14} \left( 1-same \right)$$

$$-\frac{1}{n_{23}}\sum y(1-g_{14}) same-\frac{1}{n_{23}}\sum y (1-g_{14}) \left( 1-same \right)$$

$$-\frac{1}{n_{14*}}\sum y g_{14*} same-\frac{1}{n_{14*}}\sum y g_{14*} \left( 1-same \right)$$

$$+\frac{1}{n_{23*}}\sum y(1-g_{14*}) same+\frac{1}{n_{23*}}\sum y (1-g_{14*}) \left( 1-same \right)$$

Noting that when $same=1, g_{14}=g_{14*}$, and when $same=0, g_{14}={1-g}_{14*}$, we can simplify, and group terms according to the group (A, B, C and D) they belong:

$${\Delta\hat{PB}}_{M}=$$

$$\left( \frac{1}{n_{14}}-\frac{1}{n_{14*}} \right)\sum y g_{14} same$$

$$+\left( \frac{1}{n_{23*}}-\frac{1}{n_{23}} \right)+\sum y (1-g_{14}) same$$

$$+\left( \frac{1}{n_{14}}+\frac{1}{n_{23*}} \right)\sum y g_{14} \left( 1-same \right)$$

$$-\left( \frac{1}{n_{23}}+\frac{1}{n_{14*}} \right)\sum y \left( 1-g_{14} \right)\left( 1-same \right)$$

In the above, each line corresponds to a different group, A, B, C and D.

$${\Delta\hat{PB}}_{M}=\left( \frac{1}{n_{14}}-\frac{1}{n_{14*}} \right)\sum_{A} y+\left( \frac{1}{n_{23*}}-\frac{1}{n_{23}} \right)\sum_{B} y+\left( \frac{1}{n_{14}}+\frac{1}{n_{23*}} \right)\sum_{C} y-\left( \frac{1}{n_{23}}+\frac{1}{n_{14*}} \right)\sum_{D} y$$

Thus, we have written ${\Delta\hat{PB}}_{M}$ as a sum of quantities estimated in different, non-overlapping group of patients. Calculation of variance and standard error is now straightforward

$$var\left( {\Delta\hat{PB}}_{M} \right)=\left( \frac{1}{n_{14}}-\frac{1}{n_{14*}} \right)^{2}n_{A} var\left( y | A \right)+\left( \frac{1}{n_{23*}}-\frac{1}{n_{23}} \right)^{2}+n_{B} var\left( y | B \right)+$$

$$\left( \frac{1}{n_{14}}+\frac{1}{n_{23*}} \right)^{2}n_{C} var\left( y | C \right)+\left( \frac{1}{n_{23}}+\frac{1}{n_{14*}} \right)^{2}n_{D} var\left( y | D \right)$$

where $n_{A}, n_{B}, n_{C}, n_{D}$ denote the number of patients in group A, B, C and D respectively, and variance terms can be estimated using sample variances of the observed data.

# **Estimation via matching patients one-on-one**

In addition to the methods presented in the manuscript, we hereby present an additional estimation method based on matching patients.

## ***Decision accuracy via matching***

Instead of estimating $BA_{M}$ by creating groups of similar patients as shown in Section 5.2 of the manuscript, we can estimate after matching patients one-to-one, i.e. after creating pairs of treated and untreated patients, as also proposed by Rolling and Yang.^3^ There are several approaches we can follow. We can match patients (i) according to $\hat{B}_{iM}$; (ii) using some other function of the baseline covariates; (iii) after fitting a new model in the control arm only, i.e. matching according to a prognostic score. The latter was recently proposed by Gao et al.^4^, who used a distance measure based on a random forest model for matching. These methods aim to create pairs of ‘similar’ treated and untreated patients. After matching, we compute observed and predicted benefit within each pair, and calculate percent agreement as above. Given that the number of treated and untreated patients may be different in the data, we can repeat the analysis multiple times and average, to obtain stable results. In this work, we only explored approaches (i) and (ii). One thing to note with this method is that for the case of a binary outcome, the observed benefit within a pair of patients can only take three values, 1, 0 or -1. Thus, we can only compare the sign of observed to predicted benefit for pairs where observed benefit was non-zero. Of note, the estimation of $BA_{M}$ will be sensitive to the way we match (e.g. the covariates we use and the matching algorithm). Moreover, for binary outcomes, even if we match perfectly according to $\hat{B}_{iM}$ (i.e. $B_{iM}$ is constant within each pair), the baseline risk may be different, meaning that the observed benefit may not be concordant with $\hat{B}_{iM}$. Thus, the method rests on strong assumptions, but may still be useful for comparing models.

## ***Calibration for benefit via matching (continuous outcomes only)***

We can follow the procedure described in the previous paragraph to estimate calibration for benefit after matching patients on treatment with patients on control one-on-one. The matching can be done using various methods, as noted above. After matching, we compare observed and predicted treatment benefit within each pair to estimate RMSE, intercept, slope and $R^{2}$ of the line $B_{i}\sim\hat{B}_{iM}$.

# **Details about the toy dataset shown in the main paper**

## ***Details about data generation***

To illustrate all methods of the paper we simulated a dataset of 1000 patients, 4 predictors, and a continuous outcome. We started by generating treatment assignment for each patient, as Bernoulli draws with 50% probability. Next, we generated two correlated predictors for each patient $i$, $x_{i1}$ and $x_{i2}$, by drawing from a bivariate normal distribution centered at 0 and with a variance-covariance matrix with 1 for the diagonal, 0.2 for the off-diagonal terms. Predictor $x_{i3}$ was binary, it was generated with 0.2 probability for each patient. Predictors $x_{i4}$ and $x_{i5}$ were generated by drawing from a standard normal distribution. Next, we generated the true outcome for each patient in the control treatment as

$$y_{i0}=5+0.3 x_{i1}+0.4 x_{i2}+0.3 x_{i3}+0.3 x_{i4} +e_{i}$$

$$e_{i}\sim N(0,1)$$

We generated the true treatment benefit as $B_{i}=0.5+0.3 x_{i1}- x_{i3}.$Then, the true outcome in treatment ($t=1$) was just $y_{i1}=y_{i0}+B_{i}$. Finally for each patient the observed outcome was $y_{i}=y_{i1} t_{i}+y_{i0}\left( 1-t_{i} \right)$. The generation of the dataset was done using the simcont function of the **predieval** package in R.

The distribution of the continuous predictors is shown in the following graphs.

There were 799 (201) patients with $x_{i3}=0 \left( 1 \right)$.

The mean outcome was 5.37 in the treatment, 5.00 in the control arm. The distribution of outcomes is given below:


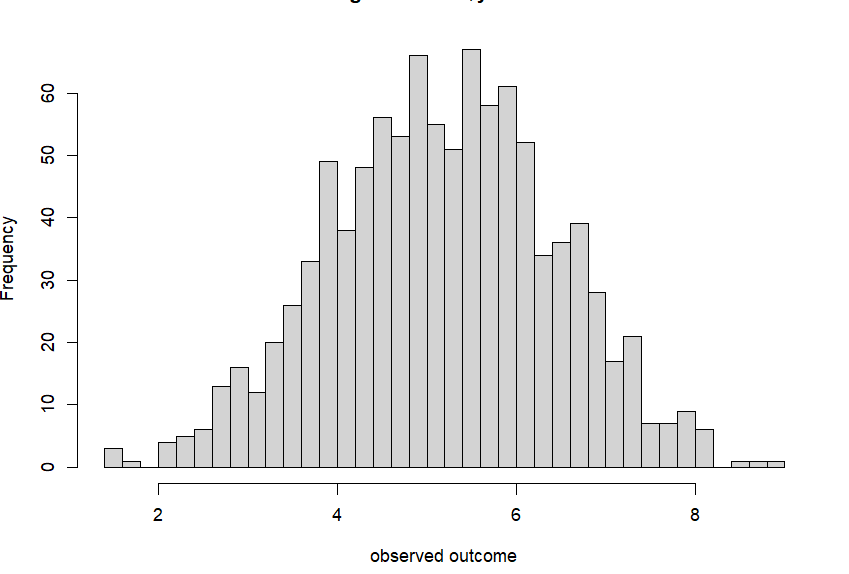


The mean true benefit in the dataset was 0.29, with 77.3% of the patients having positive treatment benefit, 22.7% negative. The distribution of true benefit is given below:

******

## ***Results from the analysis using matching methods***

Here we provide the results of using the matching method discussed in Section 3 of this Appendix. We estimated using the **predieval** package, which calls internally the **Matching** package in R^5^, to create the patient pairs. We did 500 repetitions. We estimated benefit accuracy, and results when matching by benefit were: 66% for $M_{1}$, 59% for $M_{2}$. When matching covariates by using Mahalanobis distance we found: 67% for $M_{1}$, 59% for $M_{2}$. The true values of the estimands were 95% and 79% for $M_{1}$ and $M_{2}$ respectively. Both approaches correctly suggested $M_{1}$ to be better in discrimination for benefit but underestimated the true values.

Then we turned to calibration measures. Results are shown in the table below

|  | **Model** $\boldsymbol{M}_{\boldsymbol{1}}$ | | | | **Model** $\boldsymbol{M}_{\boldsymbol{2}}$ | | | |
| --- | --- | --- | --- | --- | --- | --- | --- | --- |
| **Performance measure**  **Estimation method** | **RMSE** | $\boldsymbol{a}_{\boldsymbol{0}}$ | $\boldsymbol{a}_{\boldsymbol{1}}$ | $\boldsymbol{R}^{\boldsymbol{2}}$ | **RMSE** | $\boldsymbol{a}_{\boldsymbol{0}}$ | $\boldsymbol{a}_{\boldsymbol{1}}$ | $\boldsymbol{R}^{\boldsymbol{2}}$ |
| **True values of the estimands, estimated using 100,000 new patients** | **0.12** | **-0.05** | **1.08** | **0.95** | **0.41** | **-0.11** | **1.25** | **0.32** |
| **One-to-one matching by benefit** | 1.46 | 0.04 | 1.04 | 0.09 | 1.52 | 0.06 | 1.00 | 0.02 |
| **One-to-one matching by covariates** | 1.62 | 0.10 | 1.04 | 0.05 | 1.66 | -0.11 | 1.26 | 0.02 |

# **A simulated dataset with a binary outcome**

In addition to the example of a dataset for a continuous outcome discussed in the paper and described in the previous paragraphs in more detail, we also simulated a dataset with binary outcome

## ***Data generation***

We simulated a dataset with 2000 patients, 4 predictors and a binary outcome. We started by generating treatment assignment $t_{i}$ for each patient $i$, as Bernoulli draws with 50% probability. Next, we generated two correlated continuous predictors, $x_{i1}$ and $x_{i2}$, by drawing from a bivariate normal distribution centered at 0 and with a variance-covariance matrix with 1 for the diagonal, 0.2 for the off-diagonal terms. Predictor $x_{i3}$ was binary, it was generated with 0.2 probability for each patient. Predictor $x_{i4}$ was also binary and was generated with 0.1 probability for each patient. Below is the distribution of predictors $x_{1}$ and $x_{2}$ respectively:


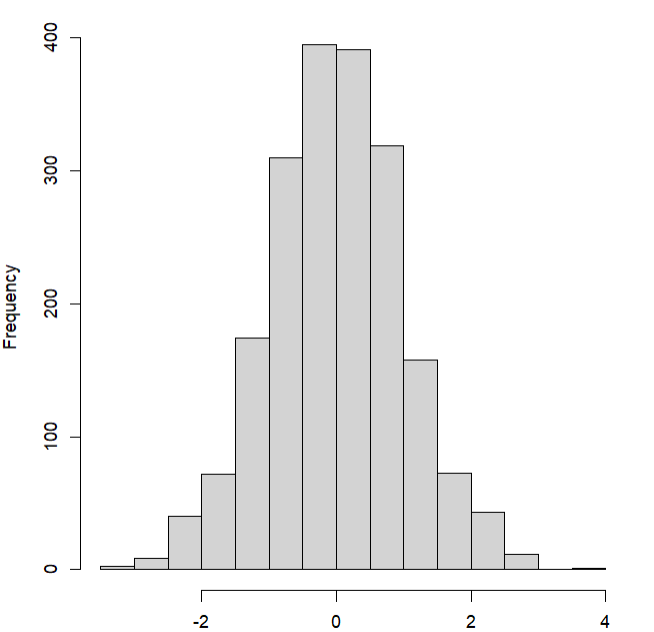

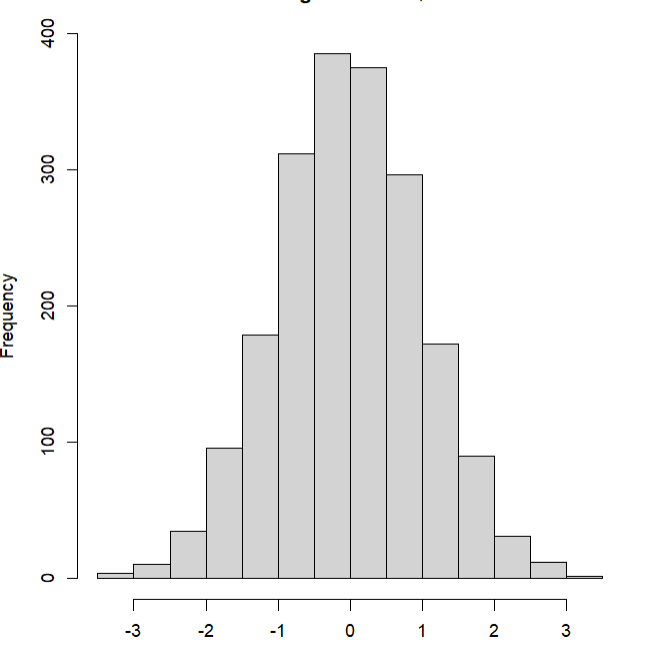


There were 392 (1608 ) patients with $x_{3}=0$ (1)$,$ and 197 (1803) patients with $x_{4}=0 (1)$.

Next, we generated the true log odds of the outcome for each patient $i$ in the control treatment as:

$${logodds}_{i0}=-2+0.5 x_{i1}+0.4 x_{i2}+0.3 x_{i3}+0.5 x_{i4}$$

We generated the true treatment benefit in the log odds ratio scale as

$$B_{i}=-0.3-0.3 x_{i1}+0.2 x_{i3}+0.1 x_{i4}$$

The true log odds in treatment ($t=1$) was generated as ${logodds}_{i1}={logodds}_{i0}+B_{i}$.

Finally for each patient the observed outcome was generated by drawing from a Bernoulli distribution with probability $p_{i}=expit({logodds}_{i1} t_{i}+{logodds}_{i0}\left( 1-t_{i} \right))$. There was a total of 275 events in the dataset, 128 in the control arm, 147 in the treatment arm. Here we assumed an event ($y_{i}=1$) to be preferable. The mean of the true benefit on the logit scale was -0.25. Below we show the distribution of the true benefit in the dataset, in the logit scale. According to the data generating mechanism, 20% of the patients in the sample would benefit (i.e. larger probability of an event) from receiving $t=1$, and 80% from $t=0$.


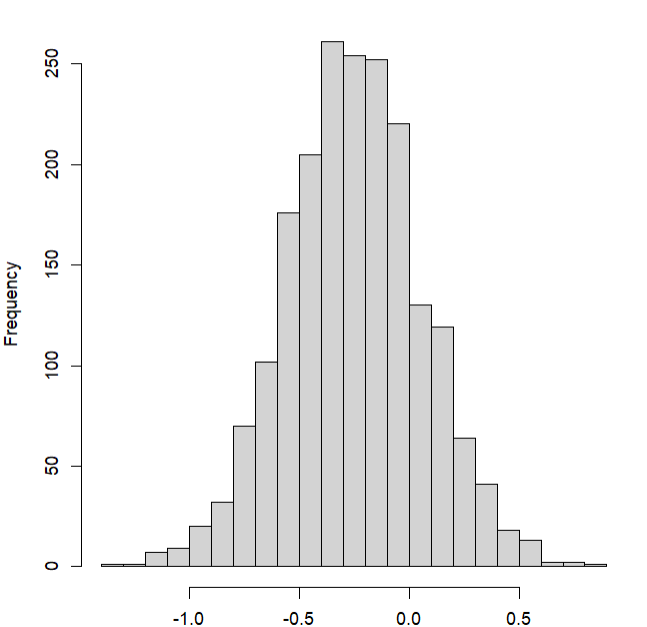


Despite the fact that the true benefit was negative, the observed mean benefit in the sample was positive: 14.4% of patients in $t=1$ experienced the event, 13.1% in the control.

The generation of the dataset was done using the simbinary function of the **predieval** package in R.

## ***Model development***

We fitted two logistic regression models, $M_{3}$: $logit\left( p(y_{i}) \right)\sim x_{i1}+t_{i}+x_{i1} t_{i}$; and $M_{4}$: $logit\left( p(y_{i}) \right)\sim x_{i1}+x_{i2}+t_{i}+x_{i2} t_{i}+x_{i4} t_{i}$. The first model was misspecified, it missed several true predictors, and included only one of the three true treatment-covariate interactions. The second model was also misspecified. It included two additional true predictors ($x_{i2}{, x}_{i3}$) but missed the two stronger true treatment-covariate interactions.

We found that $M_{4}$ performed better in predicting the outcome. The AUC was 0.68 for $M_{3}$, 0.69 for $M_{4}$. Both models predicted an average event rate of 13.5%, close to the observed value. However, when comparing the predicted treatment benefit $\hat{B}_{i}$ from each model with the true benefit $B_{i}$, we saw that $M_{4}$ performed worse. When comparing $B_{i}$ to$\hat{B}_{i}$ (in log odds ratios) we found an $RMSE=0.20$ for $M_{3}$ and 0.42 for $M_{4}$. When regressing $B_{i}\sim\hat{B}_{i}$ we found a slope of 0.91 and 0.28, and $R^{2}$ 0.92 and 0.04 respectively. This bad performance was expected since $M_{4}$ did not include any true effect modifiers. When we used the models in a new large sample of 50,000 patients generated using the same mechanism we got similar results.

Thus we see that although $M_{4}$ was better at predicting the outcome, it failed at predicting treatment benefit.

## ***Results***

We used the simulated dataset described above to fit the two models described above ($M_{3}$ and $M_{4}$). To assess their performance, we followed a 10-fold CV as per Section 1 of this appendix, repeated 100 times. The true values of the performance measures (i.e. the true external performance of the models) were calculated after using the models fitted to the whole dataset to make predictions for a large new dataset of 50,000 patients generated using the same mechanism.

We started with discrimination for benefit, assessed via the C-for-benefit (Section 4.2 in the main paper). After repeating the matching procedure 1000 times and averaging, the two models gave results 0.53 [0.49; 0.57] and 0.52 [0.47; 0.56] when matching by benefit; 0.53 [0.49; 0.58] vs. 0.50 [0.46; 0.55] when matching by covariates, for $M_{3}$ and $M_{4}$ respectively. The true values were 0.54 vs. 0.48 for $M_{3}$ and $M_{4}$ when matching by benefit or by covariates.

Then, we turned to calibration for benefit. We started with mean bias, estimated to be $0.6\%$for $M_{3}$ and $0.5\%$ for $M_{4}$. The true values of the performance measures were $-1.8\%$ and $-1.9\%$ respectively, quite far from the estimated values. This, however, was rather expected since the observed average benefit in the sample was on a different direction than the true benefit (due to the irreducible binomial error). In Figure 3 we show the calibration-for-benefit plot (i.e. grouping by $\hat{B}_{iM}$, Section 4.3.1 in main paper), for $N_{g}=5$, where it is obvious that $M_{3}$ performed better. Next, we we grouped patients into $N_{g}=5, 10$ groups according to the estimated benefit and we calculated the various measures of performance. We repeated estimation after clustering using the covariates, as in Section 4.3.2. All results are shown in Table 4. Next, we fit a regression for benefit (Section 4.3.3 in main paper). The slope of benefit was estimated at 0.79 [-0.04; 1.61]for $M_{3}$, 0.08 [-1.07; 1.21]for $M_{4}$.

Finally, we turned to decision accuracy. We estimated population benefit (Section 4.4.1, main paper), where we used 500 bootstrap iterations to obtain 95% confidence intervals. Results were $\hat{PB}_{M_{3}}=3.3\% \left[ 0.5\%; 6.0\% \right]$, and $\hat{PB}_{M_{4}}=0.8\% \left[ -2.2\%; 3.7\% \right]$, adjusted for covariates. The difference between the two, unadjusted for covariates and following the method described in Section 2.3 of this Appendix, was estimated to be 2.7% [-1.1%; 6.6%], indicating $M_{3}$ to perform better (but with large uncertainty). True values of population benefit were 2.0% and 0.1% respectively. Next, we estimated benefit accuracy. We repeated 1000 times, for $N_{g}=5, 10$. We found $\hat{BA}$ to be 65% vs 70% for $M_{3}$ and $M_{4}$ respectively, for $N_{g} = 5$. For $N_{g}=10$ results were 80% versus 60%. We also estimated benefit accuracy using the one-to-one matching method (Section 3 of this Appendix) and we found 57% vs. 51% when matching by benefit, and 56% vs. 54% when matching for covariates, for $M_{3}$ vs. $M_{4}$. Thus, we observed a marginally better performance for $M_{3}$. The true values of the performance measures were 79% for $M_{3}$ and 51% for $M_{4}$.

We conclude that most methods indicated $M_{3}$ to be better, but that estimated values were sometimes far from the true ones.

|  | **Model** $\boldsymbol{M}_{\boldsymbol{3}}$ | | | | **Model** $\boldsymbol{M}_{\boldsymbol{4}}$ | | | |
| --- | --- | --- | --- | --- | --- | --- | --- | --- |
| **Performance measure**  **Estimation method** | **RMSE** | $\boldsymbol{a}_{\boldsymbol{0}}$ | $\boldsymbol{a}_{\boldsymbol{1}}$ | $\boldsymbol{R}^{\boldsymbol{2}}$ | **RMSE** | $\boldsymbol{a}_{\boldsymbol{0}}$ | $\boldsymbol{a}_{\boldsymbol{1}}$ | $\boldsymbol{R}^{\boldsymbol{2}}$ |
| **True values of the performance measure, estimated using 50,000 new patients** | **0.20** | **-0.18** | **0.91** | **0.93** | **0.43** | **-0.26** | **0.40** | **0.04** |
| **Group by benefit** $\boldsymbol{N}_{\boldsymbol{g}}\boldsymbol{=5}$ | 0.028 | 0.00 | 0.85 | 0.22 | 0.038 | 0.01 | 0.33 | 0.04 |
| **Group by benefit** $\boldsymbol{N}_{\boldsymbol{g}}\boldsymbol{=10}$ | 0.046 | 0.00 | 0.95 | 0.39 | 0.072 | 0.01 | -0.33 | 0.01 |
| **k-means** $\boldsymbol{N}_{\boldsymbol{g}}\boldsymbol{=5}$ | 0.025 | 0.00 | 1.27 | 0.75 | 0.040 | 0.01 | 1.87 | 0.45 |
| **k-means** $\boldsymbol{N}_{\boldsymbol{g}}\boldsymbol{=10}$ | 0.031 | 0.00 | 1.17 | 0.66 | 0.046 | 0.00 | 1.48 | 0.28 |

**Table 4**: Calibration for benefit from the simulated dataset with binary outcome, comparing models $M_{3}$ and $M_{4}$ in terms of calibration for treatment benefit. Estimates obtained following a 10-fold CV repeated 100 times. Risk difference is used as an effect measure.


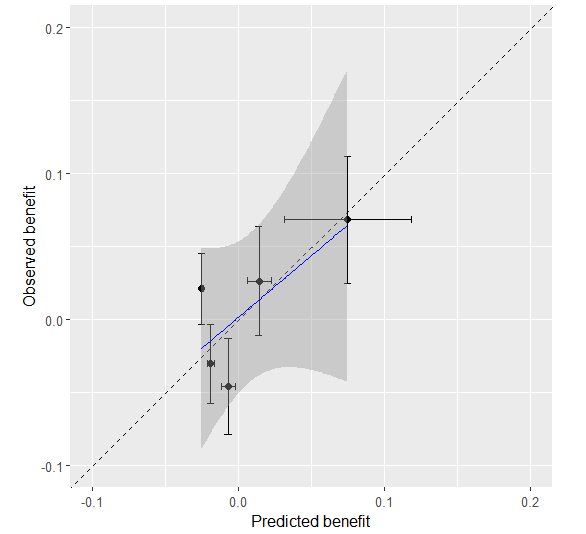

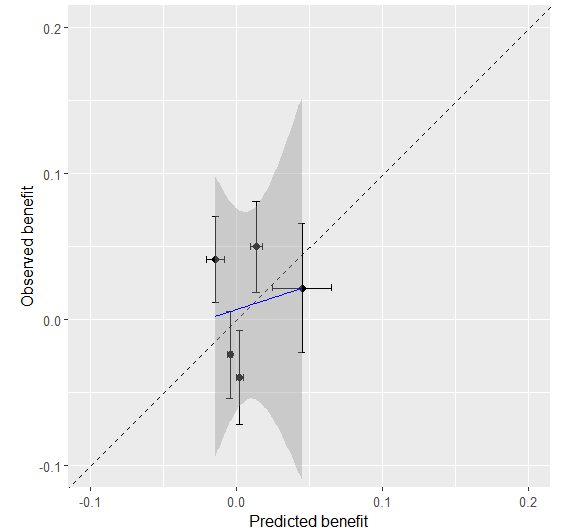


**Figure 3:** Calibration plots for benefit for models $M_{3}$ (left) and $M_{4}$ (right), on the risk difference scale, in the simulated dataset with a binary outcome.

# **Examples for using the predieval package in R**

## ***Continuous outcome***

library(devtools)

install_github("esm-ispm-unibe-ch/predieval")

library(stringr)

library(caret)

rm(list=ls()) # empty memory

set.seed(42) #the answer to life, the universe and everything

#### simulate data ----

Npat<-1000

dat<-simcont(Npat)$dat

##### 10-fold CV repeated 100 times ------

k.folds<-10

repeats<-100

dat.CV<-list()

for (k in 1:repeats){

flds <- createFolds(1:Npat, k = k.folds, list = TRUE, returnTrain = FALSE)

dat.out.CV<-list()

for (i in 1:k.folds){

dat.in.CV<-dat[-flds[[i]],]

dat.out.CV[[i]]=dat[flds[[i]],]

dat1<-dat.out.CV[[i]]; dat1$t=1

dat0<-dat.out.CV[[i]]; dat0$t=0

m1<-lm(data=dat.in.CV, y.observed~x1*t+x3*t)

dat.out.CV[[i]]$m1.CV.treat1=predict(newdata=dat1, m1)

dat.out.CV[[i]]$m1.CV.treat0=predict(newdata=dat0, m1)

m2<-lm(data=dat.in.CV,

y.observed~x1+x2+x3+x4+x1*t+x2*t)

dat.out.CV[[i]]$m2.CV.treat1=predict(newdata=dat1, m2)

dat.out.CV[[i]]$m2.CV.treat0=predict(newdata=dat0, m2)}

dat.CV[[k]]<-dat.out.CV[[1]]

for (i in 2:k.folds){dat.CV[[k]]<-rbind(dat.CV[[k]],dat.out.CV[[i]] )}

}

for(i in 1:repeats){

row.names(dat.CV[[i]])=as.numeric(str_remove(row.names(dat.CV[[i]]), "dat."))

dat.CV[[i]]<-dat.CV[[i]][order(as.numeric(row.names(dat.CV[[i]]))),]}

dat.CV.all<-dat.CV[[1]]

for(k in 2:repeats){

dat.CV.all[,c("m1.CV.treat1", "m1.CV.treat0","m2.CV.treat1", "m2.CV.treat0")]<-

dat.CV.all[,c("m1.CV.treat1", "m1.CV.treat0","m2.CV.treat1", "m2.CV.treat0")] +

dat.CV[[k]][,c("m1.CV.treat1", "m1.CV.treat0","m2.CV.treat1", "m2.CV.treat0")]

}

dat.CV.all[,c("m1.CV.treat1", "m1.CV.treat0","m2.CV.treat1", "m2.CV.treat0")]<-dat.CV.all[,c("m1.CV.treat1", "m1.CV.treat0","m2.CV.treat1", "m2.CV.treat0")]/repeats

# measures of performance for benefit

p1<-predieval(repeats=1000, Ngroups=c(10,20),

X=dat.CV.all[,c("x1", "x2","x3","x4")],

Y=dat.CV.all$y.observed,

predicted.treat.1 = dat.CV.all$m1.CV.treat1,

predicted.treat.0 = dat.CV.all$m1.CV.treat0,

treat=dat.CV.all$t, type="continuous")

p2<-predieval(repeats=1000, Ngroups=c(10,20),

X=dat.CV.all[,c("x1", "x2","x3", "x4")],

Y=dat.CV.all$y.observed,

predicted.treat.1 = dat.CV.all$m2.CV.treat1,

predicted.treat.0 = dat.CV.all$m2.CV.treat0,

treat=dat.CV.all$t, type="continuous")

# calibration plot

# Model M1

bencalibr(Ngroups=10, data=dat.CV.all, y.observed=y.observed,

predicted.treat.1 = m1.CV.treat1,

predicted.treat.0 = m1.CV.treat0, treat=t

, smoothing.function="lm", axis.limits = c(-1, 1.2))

# Model M2

bencalibr(Ngroups=10, data=dat.CV.all, y.observed=y.observed,

predicted.treat.1 = m2.CV.treat1,

predicted.treat.0 = m2.CV.treat0, treat=t

, smoothing.function="lm", axis.limits = c(-1, 1.2))

#Calculating difference in population benefit (PB) between two models

dat.CV.all$benefit.m1=dat.CV.all$m1.CV.treat1-dat.CV.all$m1.CV.treat0

dat.CV.all$benefit.m2=dat.CV.all$m2.CV.treat1-dat.CV.all$m2.CV.treat0

dat.CV.all$g14=((dat.CV.all$benefit.m1>0)& dat.CV.all$t==1 ) |((dat.CV.all$benefit.m1<0)& dat.CV.all$t==0 )

dat.CV.all$g14S=((dat.CV.all$benefit.m2>0)& dat.CV.all$t==1 ) |((dat.CV.all$benefit.m2<0)& dat.CV.all$t==0 )

dat.CV.all$same=dat.CV.all$g14==dat.CV.all$g14S

n14=sum(dat.CV.all$g14)

n23=sum(dat.CV.all$g14==F)

n14S=sum(dat.CV.all$g14S)

n23S=sum(dat.CV.all$g14S==F)

DeltaPB=with(dat.CV.all,

sum(y.observed * g14*same)*(1/n14-1/n14S)+

sum(y.observed * (1-g14)*same)*(1/n23S-1/n23)+

sum(y.observed * g14*(1-same))*(1/n14+1/n23S)-

sum(y.observed * (1- g14)*(1-same))*(1/n23+1/n14S)

)

Var.DeltaPB=

with(dat.CV.all,

sum(g14==T&same==T)*var(y.observed[g14==T&same==T])*(1/n14-1/n14S)^2+sum(g14==F&same==T)* var(y.observed[g14==F&same==T] )*(1/n23S-1/n23)^2+sum(g14==T&same==F)*var(y.observed[g14==T&same==F] )*(1/n14+1/n23S)^2+sum(g14==F&same==T)*var(y.observed[g14==F&same==F] )*(1/n23+1/n14S)^2

)

SE.DeltaPB=sqrt(Var.DeltaPB)

upper.DeltaPB=DeltaPB+1.96*SE.DeltaPB

lower.DeltaPB=DeltaPB-1.96*SE.DeltaPB

print(paste("DeltaPB: ",round(DeltaPB, digits=3), " [",round(lower.DeltaPB, digits=3), "; ",

round(upper.DeltaPB, digits=3), "]", sep=""))

## ***Binary outcome***

library(predieval)

library(stringr)

library(caret)

rm(list=ls()) # empty memory

set.seed(42) #the answer to life, the universe and everything

#### load data ----

Npat<-2000

dat<-simbinary(Npat)$dat

#### 10-fold CV repeated 100 times----

k.folds<-10

repeats<-100

dat.CV<-list()

for (k in 1:repeats){

flds <- createFolds(1:Npat, k = k.folds, list = TRUE, returnTrain = FALSE)

dat.out.CV<-list()

for (i in 1:k.folds){

dat.in.CV=dat[-flds[[i]],]

dat.out.CV[[i]]=dat[flds[[i]],]

m3<- glm(y.observed ~ x1*t, data = dat.in.CV, family = binomial)

dat1<-dat.out.CV[[i]]; dat1$t=1

dat0<-dat.out.CV[[i]]; dat0$t=0

dat.out.CV[[i]]$predict.t1.m3=predict(newdata=dat1, m3)

dat.out.CV[[i]]$predict.t0.m3=predict(newdata=dat0, m3)

m4<- glm(y.observed ~ x1+x2*t+t*x4, data = dat.in.CV, family = binomial)

dat1<-dat.out.CV[[i]]; dat1$t=1

dat0<-dat.out.CV[[i]]; dat0$t=0

dat.out.CV[[i]]$predict.t1.m4=predict(newdata=dat1, m4)

dat.out.CV[[i]]$predict.t0.m4=predict(newdata=dat0, m4)

}

dat.CV[[k]]<-dat.out.CV[[1]]

for (i in 2:k.folds){dat.CV[[k]]<-rbind(dat.CV[[k]],dat.out.CV[[i]] )}

}

for(i in 1:repeats){

row.names(dat.CV[[i]])=as.numeric(str_remove(row.names(dat.CV[[i]]), "dat."))

dat.CV[[i]]<-dat.CV[[i]][order(as.numeric(row.names(dat.CV[[i]]))),]}

dat.CV.all<-dat.CV[[1]]

for(k in 2:repeats){

dat.CV.all[,c("predict.t1.m3", "predict.t0.m3","predict.t1.m4", "predict.t0.m4")]<-

dat.CV.all[,c("predict.t1.m3", "predict.t0.m3","predict.t1.m4", "predict.t0.m4")] +

dat.CV[[k]][,c("predict.t1.m3", "predict.t0.m3","predict.t1.m4", "predict.t0.m4")]

}

dat.CV.all[,c("predict.t1.m3", "predict.t0.m3","predict.t1.m4", "predict.t0.m4")]<-

dat.CV.all[,c("predict.t1.m3", "predict.t0.m3","predict.t1.m4", "predict.t0.m4")]/repeats

# measures of performance for benefit

p3<-predieval(repeats=1000, Ngroups=c(5,10),

X=dat.CV.all[,c("x1", "x2","x3", "x4")],

Y=dat.CV.all$y.observed,

predicted.treat.1 = expit(dat.CV.all$predict.t1.m3),

predicted.treat.0 = expit(dat.CV.all$predict.t0.m3),

treat=dat.CV.all$t, type="binary", bootstraps = 500)

p4<-predieval(repeats=1000, Ngroups=c(5,10),

X=dat.CV.all[,c("x1", "x2","x3", "x4")],

Y=dat.CV.all$y.observed,

predicted.treat.1 = expit(dat.CV.all$predict.t1.m4),

predicted.treat.0 = expit(dat.CV.all$predict.t0.m4),

treat=dat.CV.all$t, type="binary", bootstraps = 500)

#calibration plot

# Model M3

bencalibr(Ngroups=4,data=dat.CV.all,

y.observed = y.observed,

predicted.treat.0 = expit(predict.t0.m3),

predicted.treat.1 = expit(predict.t1.m3),

treat = t, type="binary", axis.limits = c(-0.2, 0.2))

# Model M4

bencalibr(Ngroups=5,data=dat.CV.all,

y.observed = y.observed, predicted.treat.0 = expit(predict.t0.m4), predicted.treat.1 = expit(predict.t1.m4),

treat = t, type="binary", axis.limits = c(-0.4, 0.4))

# **References**

1. Efron, B. Bootstrap Methods: Another Look at the Jackknife. *The Annals of Statistics* **7**, 1–26 (1979).

2. Steyerberg, E. W. *et al.* Internal validation of predictive models: efficiency of some procedures for logistic regression analysis. *J Clin Epidemiol* **54**, 774–781 (2001).

3. Rolling, C. A. & Yang, Y. Model selection for estimating treatment effects. *Journal of the Royal Statistical Society: Series B (Statistical Methodology)* **76**, 749–769 (2014).

4. Gao, Z., Hastie, T. & Tibshirani, R. Assessment of heterogeneous treatment effect estimation accuracy via matching. *Stat Med* **40**, 3990–4013 (2021).

5. Sekhon, J. S. Multivariate and Propensity Score Matching Software with Automated Balance Optimization: The Matching package for R. *Journal of Statistical Software* **42**, 1–52 (2011).
